# Bye bye, linearity, bye: quantification of the mean for linear CRNs in a random environment

**DOI:** 10.1007/s00285-023-01973-x

**Published:** 2023-08-12

**Authors:** Mark Sinzger-D’Angelo, Sofia Startceva, Heinz Koeppl

**Affiliations:** 1 Electrical Engineering and Information Technology, Technische Universität Darmstadt, Darmstadt, Germany; 2 Centre for Synthetic Biology, Technische Universität Darmstadt, Darmstadt, Germany

**Keywords:** Cellular heterogeneity, Chemical reaction networks, Discrete state Markov environment, Analytic expression, Stationary mean, Zeroth-and first-order mass action kinetic, 60J28, 60K37, 92C37, 92C42

## Abstract

Molecular reactions within a cell are inherently stochastic, and cells often differ in morphological properties or interact with a heterogeneous environment. Consequently, cell populations exhibit heterogeneity both due to these intrinsic and extrinsic causes. Although state-of-the-art studies that focus on dissecting this heterogeneity use single-cell measurements, the bulk data that shows only the mean expression levels is still in routine use. The fingerprint of the heterogeneity is present also in bulk data, despite being hidden from direct measurement. In particular, this heterogeneity can affect the mean expression levels via bimolecular interactions with low-abundant environment species. We make this statement rigorous for the class of linear reaction systems that are embedded in a discrete state Markov environment. The analytic expression that we provide for the stationary mean depends on the reaction rate constants of the linear subsystem, as well as the generator and stationary distribution of the Markov environment. We demonstrate the effect of the environment on the stationary mean. Namely, we show how the heterogeneous case deviates from the quasi-steady state (Q.SS) case when the embedded system is fast compared to the environment.

## Introduction

**Fig. 1:**
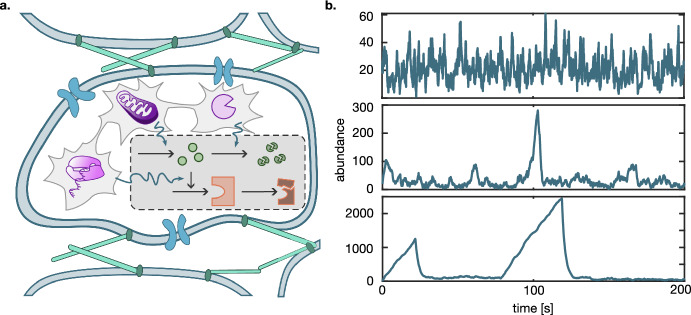
**a** The cartoon shows an example subsystem of a reaction cascade (dashed box) that is embedded into a cellular environment that includes RNA polymerase activity, ATP availability, and the concentration of a degradation enzyme. Purple environment components from left to right symbolize the RNA polymerase that modulates transcription, mitochondria that supply energy in form of ATP and proteases that actively degrade proteins. More broadly, the embedding of the single cell in a tissue and communication with neighbouring cells via the extracellular matrix (light green) or ion channels (blue) can contribute stochasticity. The reaction cascade consists of a sensor molecule (green) and an enzyme (orange) whose expression is mediated by the sensor molecule. **b** Trajectories of a birth-death-process with heterogeneous death rate $\mu$, modelled as a birth-death process on different time scales. On the fast time scale, the trajectory resembles an unmodulated birth-death process (upper panel). On the intermediate time scale, triangular excursions appear while $\mu = 0$ (middle panel). On the slow time scale, the excursions become more pronounced (lower panel). The mean death rate is the same for all cases (color figure online)

Regulation of gene expression is generally well-modelled by stochastic chemical reaction networks (CRNs) and can be successfully represented using other approaches only under certain conditions (Hahl and Kremling 2016). The modelled system, e.g., a single gene or a small gene network, exists in the context of a larger gene network and the cellular environment (Fig. 1a). It is crucial to account for the influence of this context onto the modelled system while not inflating the model complexity. A standard approach for this is to represent all external influence on a given system as a consolidated stochastic process (Zechner et al. 2014; Zechner and Koeppl 2014). In the case when the environment affects the rates of the studied process, we must consider a CRN with modulated reaction rates.

In modelling studies, protein degradation, or the death process, is often assumed to be a constant-rate process that is driven by the activity of degradation enzymes and dilution due to cell growth (Paulsson 2005). Nevertheless, the embedding environment, natural or synthetic, can regulate this process by tuning the degradation enzyme levels or the cell growth rate. In prokaryotes, protein concentrations are generally defined by the balance between production and dilution rather than degradation rates (Klumpp et al. 2009). However, tunable protein degradation can be much faster than dilution alone, and this dynamic can propagate to downstream circuits, e.g., a bistable genetic switch can change states more frequently (Cameron and Collins 2014; Huang et al. 2012). Meanwhile, in eukaryotes, protein degradation is regulated via a ubiquitin-proteasome pathway, which allows fine-tuning the degradation rates of specific proteins (Ciechanover and Schwartz 1998; Ciechanover 1998; Kornitzer and Ciechanover 2000). Further, controlling targeted protein degradation is a promising route for treating various health conditions such as cancer, neurodegenerative diseases, metabolic disorders, infections, and inflammatory diseases (Collins et al. 2017). Previously, it was not possible to perturb the protein death process in a controlled way, which hindered the detailed modeling of these processes. However, recently, new techniques for this were introduced (Collins et al. 2017; Dao and Castañeda 2020). Thus, it is now relevant and feasible to develop new modelling strategies that would incorporate modulated protein degradation rates.

The study of general chemical reaction networks dates back to the 60 s (McQuarrie 1967), after Delbrück (1940), Bartholomay (1958), Ishida (1964) and others had pioneered small case studies. Typical quantities of interest are the probability distribution or the generating function and moments both in the transient and stationary behaviour. Bartholomay (Bartholomay 1962) still emphasized the analogy of the mean equations with the deterministic counterpart. However, the following works (Gillespie 1976; McQuarrie 1967) demonstrated that bimolecular reactions cause a characteristic deviation of stochastic systems when the system is not in the thermodynamic limit, highlighting the need for other techniques. Early works computed the generating function and mean at stationarity for small bimolecular networks comprising two balanced reactions (Darvey et al. 1966). The observation that bimolecular reactions result in an unclosed mean equation brought more attention to unclosed (hierarchical) moment equations. Unclosed moment equations have prompted researchers to pursue several strategies. Among them, stochastic simulations for systems with highly abundant components are time-consuming and therefore computationally prohibitive. Equally prohibitive is the use of the master equation, which gave rise to more efficient hybrid methods (Hasenauer et al. 2014; Jahnke 2011; Menz et al. 2012). Overall, previous studies developed and extensively explored various moment closure schemes (Bronstein and Koeppl 2018; Grima 2012; Lakatos et al. 2015; Schnoerr et al. 2015), identified the limitation of moment closures with regards to bimodal distributions (Hasenauer et al. 2014) and extinction (Singh and Hespanha 2011), and pointed out the limited local character of moment closures (Kuehn 2016). More recently, linear programming under positive semi-definite constraints was employed to compute upper and lower moment bounds (Ghusinga et al. 2017; Kuntz et al. 2019; Sakurai and Hori 2017) and approximated the moments in the case of tight bounds.

Linear reaction networks are a class of CRNs whose propensities depend linearly on the state of the system, i.e., the vector of species copy numbers. Assuming mass-action kinetics, this restriction corresponds to admitting only first- and zeroth-order reactions. For this class, the first- and second order moment dynamics are well-known both in the transient and stationary phase (Warren et al. 2006). In particular, the stationary mean of the stochastic system equals the equilibrium concentration of the corresponding deterministic system (Anderson and Kurtz 2015). We have a clear understanding of the case when a linear subsystem is modulated in its zeroth-order reactions. Several research groups characterized the second-order moments (Gupta and Khammash 2022; Raj et al. 2006; Warren et al. 2006) for this case and Zechner and Koeppl (2014) introduced a method with which they (approximately) simulated marginal subsystem trajectories for various example networks. Zeroth-order modulation by a linear environment is included in the theory on linear systems, because the joint subsystem-environment network is linear. Non-linear reaction systems were embedded in a linear environment while assuming linear subsystem-environment interaction (Falk et al. 2019), i.e., zeroth-order modulation (in both directions).

The effect of a random environment on the noise in a subsystem, i.e., on the variance, is well-studied. Many research groups adopted the decomposition of noise into intrinsic and extrinsic components since its introduction in the seminal paper (Swain et al. 2002), and this concept was expanded upon in later studies (Bowsher and Swain 2012; Hilfinger et al. 2012; Gupta and Khammash 2022; Tostevin et al. 2012). Of equal interest is the question how the environment shapes the stationary distribution of the subsystem, e.g., bimodality (Holehouse et al. 2020). In contrast, we investigate the effect of the random environment on the mean of a subsystem species. This effect is visible when first-order reactions are modulated as the following simple example illustrates. Consider a birth-death process with birth rate $\lambda$ and death rate $\mu$ whose mean approaches $\lambda /\mu$ in the equilibrium. Zero-order modulation corresponds to a modulation in the birth rate, i.e., stochastic $\lambda$ with mean ${\bar{\lambda }}$. By the linearity of the function $\lambda \mapsto \lambda /\mu$ and the linearity of the mean, the heterogeneous mean $\bar{\lambda }/\mu$ does not reflect the heterogeneity at the mean level. However, if $\mu$ is stochastic, the non-linearity of $\mu \mapsto \lambda /\mu$ invalidates this argument. Instead, the excursions in the abundance that emerge during $\mu = 0$ can distort the heterogeneous mean away from $\lambda /{\bar{\mu }}$ (Fig. 1b). In the experimental context, this means that even bulk data carries the fingerprint of a heterogeneous environment.

The effect of a modulated degradation rate on the mean was studied in a gene expression model with a two-state promoter, with and without negative feedback, for a lognormally distributed extrinsic noise (Keizer et al. 2019). The authors confined their study to a slow extrinsic noise, used a linear noise approximation and a slow noise expansion. The simple birth-death process in a random environment has been studied in queuing theory. It corresponds to an $M/M/\infty$ queue, i.e., a queuing system with infinitely many servers and exponential arrival and serving times. The birth rate is the rate of arrival, while the death rate corresponds to the service rate. For a Markov environment, the stationary distribution, stationary factorial moments, and the transient evolution equation of the moments were derived in O’Cinneide and Purdue (1986). Bimodal stationary distribution was reported. Complementary to this result, the special case of the random telegraph modulated service rates was fully characterized in Baykal-Gursoy and Xiao (2004). It was extended to simultaneous birth- and death-modulation by a semi-Markov environment in Falin (2008).

In this work, we consider the general class of linear reaction systems (subsystems) whose reaction rates are modulated by a discrete state Markov environment. As our main contribution, we analytically express the stationary mean for this class of CRNs in terms of the subsystem rate constants, the environment generator matrix and the environment equilibrium distribution. This extends the existing results in queuing theory to more reaction channels than the birth-death channel. It also provides an exact alternative, at least on the mean level, to various approximations that have been used to understand the effect of extrinsic noise on the mean, variance, power spectrum and distribution. Furthermore, we propose a new method that quantifies the shares that the environment states contribute to the stationary mean of a subsystem species. Finally, we analyze the deviation from the Q.SS behaviour in a variety of case studies to illustrate several non-linear effects of the random environment.

## Model class

### General CRN model

A CRN is defined as a system of $M$ chemical reactions ${{\,\textrm{R}\,}}_1, \dots , {{\,\textrm{R}\,}}_M$ involving $d$ reactant species ${{\textbf {X}}}= [X_1, \dots , X_d]$ with the stoichiometric substrate and product coefficients $S_{ij}$ and $P_{ij}$, respectively, and reaction rates $c_j$, where $i \in \{1, \dots , d\}$, $j \in \{1, \dots , M\}$:

$$\begin{aligned} \begin{matrix} {{\,\textrm{R}\,}}_1:&{}S_{11} X_1 + \cdots + S_{d1} X_d&{} \overset{c_1}{\longrightarrow }\ {} &{} P_{11} X_1 + \cdots + P_{d1} X_d\\ \vdots &{}&{}\vdots &{}\\ {{\,\textrm{R}\,}}_M:&{}S_{1M} X_1 + \cdots + S_{dM} X_d&{} \overset{c_M}{\longrightarrow }\ {} &{} P_{1M} X_1 + \cdots + P_{dM} X_d. \end{matrix} \end{aligned}$$

The propensities of the system transitioning between states follow the mass-action kinetics, with the propensities $\vec {{f}} = [{f}_1,\dots ,{f}_M]$ being a function of the stoichiometric constants and the reaction rates, where the function form depends on the values of the stoichiometric constants. Note that reactions of higher than the second order, i.e., reactions with $\sum _{i=1}^{d}S_{ij} > 2$, are generally not considered, as the probability of such reactions occurring is negligibly low compared to the reactions of the lower orders.

### Homogeneous linear CRN

Consider a *linear* CRN with the vector ${{\textbf {X}}}_t \in {\mathbb {N}}^d$ of species counts, namely, a CRN where the vector of propensities for its $M$ reactions is of the form

1 $$\begin{aligned} \vec {{f}}: {\mathbb {N}}^d\rightarrow {\mathbb {R}}_{\ge 0}^M, \vec {{f}}({\textbf{x}}) = {{\varvec{\Gamma }}}{\textbf{x}}+ {{{\gamma }}}. \end{aligned}$$

If all reactions follow mass-action kinetics, this implies that only zeroth or first order reactions are admissible (Warren et al. 2006). Then ${{\varvec{\Gamma }}}{\textbf{x}}$ and ${{{\gamma }}}$ are the vectors with the propensities of the first-order and the zero-order reactions, respectively. Next, we construct the stoichiometric matrix that stores the change vectors for each reaction:

$$\begin{aligned} {\textbf{N}}= \begin{bmatrix}\vert &{} &{}\vert \\ \vec {{\nu }}_1&{} \dots &{} \vec {{\nu }}_M\\ \vert &{} &{}\vert \end{bmatrix} \in {\mathbb {Z}}^{d\times M} \end{aligned}$$

via $N_{ij} = P_{ij} - S_{ij}$ and $\vec {{\nu }}_j = {{p}_{\cdot j}} - {{s}_{\cdot j}}$. This allows to write down the evolution of the mean:

2 $$\begin{aligned} \frac{\,\mathrm d}{\,\mathrm dt} {\mathbb {E}}[{{\textbf {X}}}_t] = {\mathbb {E}}\left[ \sum _{j = 1}^M\vec {{\nu }}_j {f}_j({{\textbf {X}}}_t)\right] = {\textbf{N}}\vec {{f}}({\mathbb {E}}[{{\textbf {X}}}_t]) = {\textbf{N}}{{\varvec{\Gamma }}}{\mathbb {E}}[{{\textbf {X}}}_t] + {\textbf{N}}{{{\gamma }}}=: {{\textbf {b}}}- {{\textbf {A}}}{\mathbb {E}}[{{\textbf {X}}}_t]. \nonumber \\ \end{aligned}$$

The matrix ${{\textbf {A}}}= -{\textbf{N}}{{\varvec{\Gamma }}}$ captures the first-order reaction rates, while the vector ${{\textbf {b}}}= {\textbf{N}}{{{\gamma }}}$ accounts for the zero-order reaction rates. Throughout this paper, we assume that in the homogeneous case ${{\textbf {A}}}$ has only eigenvalues with positive real part. This property is called Hurwitz-stable, and it guarantees the ergodicity of the system, in particular the existence of and convergence to the stationary mean (Gupta et al. 2014).

### Linear CRN in a random environment

We have just described the homogeneous case. Next, we embed the linear chemical reaction network in a random environment. We assume that ${Z}$ is a stationary continuous-time Markov chain (CTMC) on a discrete state space ${\mathcal {Z}}$. The environment modulates ${{\textbf {X}}}$ by ${Z}$-dependent propensities which replace Eq. (1) by

3 $$\begin{aligned} \vec {{f}}({\textbf{x}}\vert {z}) = {{\varvec{\Gamma }}}({z}) {\textbf{x}}+ {{{\gamma }}}({z}) \end{aligned}$$

with corresponding family of matrices ${{\textbf {A}}}({z})$ and vectors ${{\textbf {b}}}({z})$ as defined in Eq. (2). We refer to such a linear CRN in a random environment also as a heterogeneous, or modulated, linear CRN. Throughout this paper, we assume that ${{\textbf {A}}}({z})$ has only eigenvalues with non-negative real part for each ${z}\in {\mathcal {Z}}$. In contrast to the homogeneous case, 0 may be included as an eigenvalue. This assumption is needed for the proof of proposition 2 and for the evaluation in Eq. (20) to prove the main theorem 3. Both proofs are based on the Lemma 4, see Appendix A.2, that uses the assumption.

Most prominently, ${{\textbf {Z}}}$ can be a CRN on the state space of (environmental) species counts ${\mathcal {Z}}\subseteq {\mathbb {N}}^m$ (Liebermeister et al. 2005; Zechner and Koeppl 2014). In this case, we call ${{\textbf {X}}}$ the *subsystem* of the joint reaction network $({{\textbf {X}}}, {{\textbf {Z}}})$. The equation that governs the mean now reads

4 $$\begin{aligned} \frac{\,\mathrm d}{\,\mathrm dt} {\mathbb {E}}[{{\textbf {X}}}_t] = {\mathbb {E}}[{{\textbf {b}}}({{\textbf {Z}}}_t)] -{\mathbb {E}}[{{\textbf {A}}}({{\textbf {Z}}}_t){{\textbf {X}}}_t], \end{aligned}$$

involving non-linear modulation terms ${\mathbb {E}}[{{\textbf {A}}}({{\textbf {Z}}}_t){{\textbf {X}}}_t]$.

### Quasi-steady state reference model

With a quasi-steady state (Q.SS) assumption on the environment, we aim to map a heterogeneous linear CRN to a homogeneous one. To this end, we replace the subsystem propensities $\vec {{f}}({\textbf{x}}\vert {z})$ by ${{\bar{{f}}}}({\textbf{x}}) = {\mathbb {E}}[\vec {{{{\gamma }}}}({z})] + {\mathbb {E}}[{{\varvec{\Gamma }}}({z})]{\textbf{x}}$. Even though the quasi-steady state method has seen applications with much less coarse approximations in stochastic models, i.e., at the level of the master equation (Rao and Arkin 2003), we use the term in the described way at the mean level. The corresponding first-order rate matrix and zero-order rate vector, see Sect. 2.2, are ${\bar{{{\textbf {A}}}}} = -{\textbf{N}}{\mathbb {E}}[{{\varvec{\Gamma }}}({z})], {\bar{{{\textbf {b}}}}} = {\textbf{N}}{\mathbb {E}}[{{{\gamma }}}({z})]$. In the case of ${\mathcal {Z}}\subseteq {\mathbb {R}}_{\ge 0}^{m}$ and if ${{\varvec{\Gamma }}}$ or ${{{\gamma }}}$ depend linearly on ${{\textbf {z}}}$, then ${{\bar{{{\textbf {A}}}}}} = -{\textbf{N}}{{\varvec{\Gamma }}}({{\bar{{{\textbf {z}}}}}})$ or ${\bar{{{\textbf {b}}}}} = {\textbf{N}}{{{\gamma }}}({{\bar{{{\textbf {z}}}}}})$, respectively. The mean Eq. (4) reads

5 $$\begin{aligned} \frac{\,\mathrm d}{\,\mathrm dt} {\mathbb {E}}[{{\textbf {X}}}_t] = {\bar{{{\textbf {b}}}}} - {\bar{{{\textbf {A}}}}} {\mathbb {E}}[{{\textbf {X}}}_t] \end{aligned}$$

and converges to the equilibrium

6 $$\begin{aligned} {\mathbb {E}}[{{\textbf {X}}}^{Q.SS}_\infty ] = {\bar{{{\textbf {A}}}}}^{-1}{\bar{{{\textbf {b}}}}}. \end{aligned}$$

We use the Q.SS model as a reference model. The deviation of the heterogeneous from the homogeneous reference model quantifies the effect of the random environment on the subsystem. Quasi-steady state model reduction is typically justified by the assumption that the environment operates on a faster time scale than the subsystem. This particular rather coarse Q.SS model cannot be justified as a model reduction technique, when taking into consideration that more elaborated and superior techniques (Cao et al. 2005; Mastny et al. 2007; Rao and Arkin 2003) were developed. However, we justify our Q.SS in the reverse perspective. In the modeling context, when we build a hierarchy of models, we may start with homogeneous reaction rates, i.e., a deterministic constant environment. As we pass to heterogeneous rates, the rate means are kept constant, e.g., if the environment means were obtained from separate measurements.

Then the heterogeneous case deviates from the homogeneous Q.SS case. Is it necessary to include a higher-order moment analysis in order to detect the deviation? We demonstrate how the fingerprint of the heterogeneous rates emerges already in the stationary mean of the subsystem.

As a point of departure, we first portray a base case without deviation at the mean level, i.e., the stationary mean of the Q.SS model coincides with the heterogeneous stationary mean. Suppose, the environment modulates the subsystem only via zero-order reactions, i.e. ${{\textbf {A}}}({z}) \equiv {\bar{{{\textbf {A}}}}} = {{\textbf {A}}}$ is independent of the environment, while ${{\textbf {b}}}({z})$ may maintain dependencies. Then Eq. (4) reduces to

$$\begin{aligned} \frac{\,\mathrm d}{\,\mathrm dt} {\mathbb {E}}[{{\textbf {X}}}_t] = {\mathbb {E}}[{{\textbf {b}}}({z})] - {{\textbf {A}}}{\mathbb {E}}[{{\textbf {X}}}_t] \end{aligned}$$

with equilibrium state ${\mathbb {E}}[{{\textbf {X}}}_\infty ] = {{\textbf {A}}}^{-1}{\mathbb {E}}[{{\textbf {b}}}({z})] = {\mathbb {E}}[{{\textbf {X}}}^{Q.SS}_\infty ]$. Only in the second (and higher) order moments the exact network deviates from the Q.SS model (Gupta and Khammash 2022). The additional term which enters in the variance expressions is commonly interpreted as extrinsic noise, opposed to the intrinsic fluctuations that are attributed to the subsystem alone (Tostevin et al. 2012).

While the stationary mean is not affected by the zero-order modulation, this is much different when we allow modulation of first-order reactions. The question how the random environment affects the subsystem even on the mean level guides the remainder of this paper. Since the analytic expression for the stationary mean of the heterogeneous system that we provide in Eqs. (10), (13) and (14) below is complex, we mainly address this question numerically in the case studies, Sect. 4. However, in the case of a birth-death process with modulated degradation, an analytical answer can be obtained. We provide it in Eq. (23) below for a two-state environment and more generally in Sect. 4.1.4.

## Results

We analytically express the stationary mean for the class of CRNs introduced in the previous section. The expressions only depend on the subsystem rate constants and standard characteristics of the Markov environment that we specify as follows. Denote by ${\Lambda }({z}', {z})_{{z}', {z}\in {\mathcal {Z}}}$ its generator and by ${\pi }({z})$ its stationary distribution, i.e., ${{\varvec{\Lambda }}}{\pi } = 0$. Introduce the notation ${\Lambda }_0({z}):= - {\Lambda }({z}, {z}) = \sum _{{z}'\ne {z}} {\Lambda }({z}', {z})$ for the total exit rate. Let $(\tau _n)_{n \in {\mathbb {N}}}$ be the jump times of ${Z}$. These induce the discrete-time Markov chain $({W}_n)_n:= ({Z}(\tau _n))_n$ with transition kernel

$$\begin{aligned} K({z}', {z}) = {\left\{ \begin{array}{ll}\frac{{\Lambda }({z}', {z})}{{\Lambda }_0({z})},&{} {z}' \ne {z}\\ 0, &{}{z}' = {z}\end{array}\right. }. \end{aligned}$$

The process ${W}_n$ is called embedded discrete-time Markov chain that corresponds to the jump epochs of ${Z}(t)$ (Blom et al. 2014).

### Proposition 1

Let ${W}$ be the embedded chain of a Markov jump process on ${\mathcal {Z}}$ with stationary distribution $\pi ({z})$ and total exit rates ${\Lambda }_0({z})$, ${z}\in {\mathcal {Z}}$. Define $\tilde{{\pi }}({z}):= {\pi }({z}){\Lambda }_0({z})$. Then $\tilde{{\pi }}$ satisfies the stationarity condition for the embedded chain, i.e., is an unnormalized stationary distribution of ${W}$.

### Proof

For the check of the stationarity condition, see Appendix A.1. $\square$

### Auxiliary machinery

For the computation of the stationary mean, we use the average values of the process ${{\textbf {X}}}$ at the end of intervals $[\tau _n, \tau _{n+1}]$ in environmental state ${z}$. The computation of these average values is facilitated by the fact that, conditional on the environment, the subsystem expectation progresses linearly.

#### Definition 1

Define $${\textbf{x}}(n, {z}):= {\mathbb {E}}[{{\textbf {X}}}(\tau _{n+1})\vert {Z}(\tau _n) = {z}]$$ and $${{\textbf {Y}}}(t):= {\mathbb {E}}[{{\textbf {X}}}(t)\vert {Z}_{[0,t]}]$$.

Conditional on the history of ${Z}$, the subsystem ${{\textbf {X}}}$ is linear and hence ${{\textbf {Y}}}$ evolves like

7 $$\begin{aligned} \frac{\,\mathrm d}{\,\mathrm dt} {{\textbf {Y}}}(t) = {{\textbf {b}}}({Z}(\tau _n)) - {{\textbf {A}}}({Z}(\tau _n)){{\textbf {Y}}}(t), \quad \tau _n \le t \le \tau _{n+1}. \end{aligned}$$

We note that the value ${Z}(\tau _n)$ is the only information in the history of the environment that governs the evolution (locally in time). For the value ${{\textbf {Y}}}(t)$ global in time, the initial jump value ${{\textbf {Y}}}(\tau _n)$ is needed, which depends in a more complex way on past values of ${Z}$. This is solved interval-wise by

8 $$\begin{aligned} {{\textbf {Y}}}(t) = e^{-{{\textbf {A}}}({z})(t-t_0)}{{\textbf {Y}}}(t_0) + \int _{t_0}^t e^{-{{\textbf {A}}}({z})(s-t_0)}\,\mathrm ds \; {{\textbf {b}}}({z}). \end{aligned}$$

Choosing $t_0 = \tau _n$ and $t = \tau _{n+1}$, we just expressed

9 $$\begin{aligned} ({{\textbf {Y}}}(\tau _{n+1}), {Z}(\tau _{n+1})) = g({{\textbf {Y}}}(\tau _n), {Z}(\tau _n), \tau _{n+1}-\tau _n) \end{aligned}$$

for a deterministic update function *g*. By the update characterization of Markov chains, the independent waiting times $(\tau _{n+1}-\tau _n)_n$ and independence of $\tau _{n+1} - \tau _{n}$ from $({{\textbf {Y}}}(\tau _n), {Z}(\tau _n))$, we get that the pair $({{\textbf {Y}}}(\tau _n),{Z}(\tau _n))_n$ forms a Markov chain. Furthermore, by the tower property of conditional expectations, we obtain the following link between ${\textbf{x}}$ and ${{\textbf {Y}}}$

$$\begin{aligned} {\textbf{x}}(n, {z}) = {\mathbb {E}}[{{\textbf {Y}}}(\tau _{n+1})\vert {Z}(\tau _n) = {z}]. \end{aligned}$$

At stationarity, ${\textbf{x}}(n, {z})$ does not depend on *n* anymore, because $({{\textbf {Y}}}(\tau _{n+1}), {Z}(\tau _n))$ has the same distribution as $({{\textbf {Y}}}(\tau _{n}), {Z}(\tau _{n-1}))$. Hence, we can drop *n*. More formally:

#### Definition 2

Define $({\tilde{{{\textbf {Y}}}}}_n, {\tilde{{Z}}}_n)_n$ as the stationary version of the Markov chain $({{\textbf {Y}}}(\tau _n),{Z}(\tau _n))_n$ and ${\tilde{{\textbf{x}}}}(n, {z}):= {\mathbb {E}}[{\tilde{{{\textbf {Y}}}}}_{n+1}\vert {\tilde{{Z}}}_n = {z}]$. Define ${\textbf{x}}({z}):= {\tilde{{\textbf{x}}}}(1, {z})$.

#### Proposition 2

The ${\textbf{x}}({z})$, defined in Definition 2, satisfy the linear equations

10 $$\begin{aligned} {{\textbf {A}}}({z}){\textbf{x}}({z}) = \sum _{{z}' \in {\mathcal {Z}}} {\Lambda }({z}, {z}')\frac{{\pi }({z}')}{{\pi }({z})} {\textbf{x}}({z}') + {{\textbf {b}}}({z}). \end{aligned}$$

Suppose that ${\pi }({z})$ satisfies detailed balance, then

11 $$\begin{aligned} {{\textbf {A}}}({z}){\textbf{x}}({z}) = ({{\varvec{\Lambda }}}^T{\textbf{x}})({z}) + {{\textbf {b}}}({z}). \end{aligned}$$

#### Proof

See Appendix A.2. $$\square $$

The structure of Eq. (10) can be captured graphically. Let us visualize the recursion by a directed graph on the set of nodes ${\mathcal {Z}}$. There is an edge from ${z}'$ to ${z}$, if ${\textbf{x}}({z}')$ has a non-zero coefficient for the equation of ${\textbf{x}}({z})$. Then this graph is precisely the transition graph of the Markov chain ${Z}$. In particular the sparsity of Eq. (10) is dictated by the sparsity of ${{\varvec{\Lambda }}}$.

#### Remark 1

Define ${\textbf{y}}({z}):= {\pi }({z}){\textbf{x}}({z})$ and denote by $\mathbf {{\textbf{y}}}^T = [{\textbf{y}}({z}_0)^T, {\textbf{y}}({z}_1)^T, \dots ]$ the concatenated vector for an enumeration of the environment state. Further define the block matrices

$$\begin{aligned} \mathbf {{{\textbf {A}}}} = \begin{bmatrix} {{\textbf {A}}}({z}_0) &{} {\textbf{0}} &{} \cdots \\ {\textbf{0}} &{}{{\textbf {A}}}({z}_1) &{}\\ \vdots &{}&{}\ddots \end{bmatrix} \in {\mathbb {R}}^{\vert {\mathcal {Z}}\vert d\times \vert {\mathcal {Z}}\vert d}, \mathbf {{{\textbf {B}}}} = \begin{bmatrix} {{\textbf {b}}}({z}_0) &{} {\textbf{0}} &{} \cdots \\ {\textbf{0}} &{}{{\textbf {b}}}({z}_1) &{}\\ \vdots &{}&{}\ddots \end{bmatrix} \in {\mathbb {R}}^{\vert {\mathcal {Z}}\vert d\times \vert {\mathcal {Z}}\vert }. \end{aligned}$$

Then Eq. (10) can be written as

12 $$\begin{aligned} (\mathbf {{{\textbf {A}}}} - {{\varvec{\Lambda }}}\otimes {{\,\mathrm{{\textbf{I}}}\,}}_d)\mathbf {{\textbf{y}}} = \mathbf {{{\textbf {B}}}}{{\pi }} \end{aligned}$$

where ${{\,\mathrm{{\textbf{I}}}\,}}_d$ is the $(d\times d)$ identity matrix, $\otimes$ is the Kronecker- or tensor-product and ${\pi } \in {\mathbb {R}}^{\vert {\mathcal {Z}}\vert \times 1}$ is the stationary probability vector. Denote by $\mathbf {{\textbf{x}}}^T = [{\textbf{x}}({z}_0)^T, {\textbf{x}}({z}_1)^T, \dots ]$ the concatenated vector for an enumeration of the environment state. Furthermore consider $\mathbf {{{\textbf {b}}}} = \mathbf {{{\textbf {B}}}} {\textbf{e}}$ with ${\textbf{e}}\in {\mathbb {R}}^{ \vert {\mathcal {Z}}\vert \times 1}$ the vector with ones in all entries. Then Eq. (11) can be written as

$$\begin{aligned} (\mathbf {{{\textbf {A}}}} - {{\varvec{\Lambda }}}^T\otimes {{\,\mathrm{{\textbf{I}}}\,}}_d)\mathbf {{\textbf{x}}} = \mathbf {{{\textbf {b}}}}. \end{aligned}$$

### Exact stationary mean evaluation (ESME)

We computed the average values of the process ${{\textbf {X}}}$ at the end of intervals $[\tau _n, \tau _{n+1}]$ with environmental ’label’ ${z}$. With these auxiliary quantities, we obtain the following main result for the stationary mean.

#### Theorem 3

(ESME) Let ${{\textbf {X}}}$ be a linear CRN in a random environment as described in Sect. 2.3. With ${\textbf{x}}$ defined as in Definition (2) and ${\pi }$ the stationary distribution of ${Z}$, it holds

13 $$\begin{aligned} {\mathbb {E}}[{{\textbf {X}}}_\infty ] = \sum _{{z}\in {\mathcal {Z}}} {\pi }({z}){\textbf{x}}({z}) \end{aligned}$$

#### Proof

See next section and Appendix A.2. $\square$

#### Remark 2

If we invest Eq. (12), the resulting Eq. (13) for ESME is rewritten in matrix–vector notation

14 $$\begin{aligned} {\mathbb {E}}[{{\textbf {X}}}_\infty ] = ({\textbf{e}}^T \otimes {{\,\mathrm{{\textbf{I}}}\,}}_d) (\mathbf {{{\textbf {A}}}} - {{\varvec{\Lambda }}}\otimes {{\,\mathrm{{\textbf{I}}}\,}}_d)^{-1} \mathbf {{{\textbf {B}}}}{{{{\pi }}}} \end{aligned}$$

with $\otimes$ the Kronecker-product, ${{\,\mathrm{{\textbf{I}}}\,}}_d$ the $d\times d$-identity matrix and ${\textbf{e}}\in {\mathbb {R}}^{ \vert {\mathcal {Z}}\vert \times 1}$ the vector with ones in all entries. For $d= 1$, the expression reduces to

$$\begin{aligned} {\textbf{e}}^T (\mathbf {{{\textbf {A}}}} - {\Lambda })^{-1} \mathbf {{{\textbf {B}}}}{{\pi }} \end{aligned}$$

for diagonal matrices $\mathbf {{{\textbf {A}}}}, \mathbf {{{\textbf {B}}}}$. This is in agreement with the expression obtained in (O’Cinneide and Purdue 1986, theorem 3.1). If ${\pi }({z})$ satisfies detailed balance, then it also holds

15 $$\begin{aligned} {\mathbb {E}}[{{\textbf {X}}}_\infty ] = ({\pi }^T \otimes {{\,\mathrm{{\textbf{I}}}\,}}_d) (\mathbf {{{\textbf {A}}}} - {{\varvec{\Lambda }}}^T\otimes {{\,\mathrm{{\textbf{I}}}\,}}_d)^{-1} \mathbf {{{\textbf {b}}}}. \end{aligned}$$

### Proof of the ESME expression

In this section, we provide the essential part of the proof for the main theorem 3 (ESME) in detail. This equips the reader with the intuition on quantifying the shares that environmental states contribute to the stationary mean. We define these in the next section. Note that our proof deviates from those in queuing theory in that it does not pursue a generating function approach. For technical parts of the proof we refer to Appendix A.2.

By the ergodic theorem, we can move from the ensemble mean to the temporal mean

16 $$\begin{aligned} {\mathbb {E}}[{{\textbf {X}}}_\infty ] = \lim _{N \rightarrow \infty }\frac{1}{\tau _N}\int _0^{\tau _N} {{\textbf {X}}}(t)\,\mathrm dt. \end{aligned}$$

Next, we partition the time axis at the jump times $\tau _n$ both in the numerator and denominator to obtain

17 $$\begin{aligned} {\mathbb {E}}[{{\textbf {X}}}_\infty ] = \lim _{N \rightarrow \infty }\frac{1}{\sum _{n = 0}^{N-1} (\tau _{n+1}-\tau _n)}\sum _{n = 0}^{N-1}\int _{\tau _{n}}^{\tau _{n+1}} {{\textbf {X}}}(t)\,\mathrm dt. \end{aligned}$$

The summands (in both sums) are ordered by their appearance in time. The idea is now to sort them by the values of ${Z}(\tau _n)$. This is achieved by multiplying each summand with unity of the form $\sum _{{z}\in {\mathcal {Z}}} \mathbb {1}({Z}(\tau _n) = {z})$ and changing the order of summation. The outer sum is now indexed by ${z}\in {\mathcal {Z}}$. The next idea is to normalize the numerator and denominator by *N* to get empirical averages. As $N \rightarrow \infty$, the ergodic theorem allows us to move back to expectations. In the denominator, this reveals the average waiting time as a mixture of inverse exit rates:

$$\begin{aligned}&\sum _{{z}\in {\mathcal {Z}}}\lim _{N \rightarrow \infty } \frac{1}{N} \sum _{n = 0}^{N-1} \mathbb {1}({Z}(\tau _n) = {z})(\tau _{n+1}-\tau _n) \\&\quad = \sum _{{z}\in {\mathcal {Z}}} {\mathbb {P}}[{Z}(\tau _n) = {z}]{\mathbb {E}}[\tau _{n+1} - \tau _n\vert {Z}(\tau _n) = {z}]\\&\quad = \sum _{{z}\in {\mathcal {Z}}} {\mathbb {P}}[{Z}(\tau _n) = {z}] {\Lambda }_0({z})^{-1}. \end{aligned}$$

In the numerator, the contributions of ${Z}(\tau _n) = {z}$ to the sum is handled analogously by moving from the empirical mean to the expectation

18 $$\begin{aligned}&\sum _{{z}\in {\mathcal {Z}}} \lim _{N \rightarrow \infty }\frac{1}{N} \sum _{n = 0}^{N-1}\mathbb {1}({Z}(\tau _n) = {z}) \int _{\tau _n}^{\tau _{n+1}} {{\textbf {X}}}(t)\,\mathrm dt \nonumber \\&\quad = \sum _{{z}\in {\mathcal {Z}}}{\mathbb {E}}\left[ \mathbb {1}({Z}(\tau _n) = {z})\int _{\tau _n}^{\tau _{n+1}} {{\textbf {X}}}(t)\,\mathrm dt \right] \end{aligned}$$

19 $$\begin{aligned}&= \sum _{{z}\in {\mathcal {Z}}}{\mathbb {P}}[{Z}(\tau _n) = {z}]{\mathbb {E}}\left[ \int _{\tau _n}^{\tau _{n+1}} {{\textbf {X}}}(t)\,\mathrm dt \vert {Z}(\tau _n) = {z}\right] . \end{aligned}$$

The integrals in Eq. (19) evaluate to

20 $$\begin{aligned} {\mathbb {E}}\left[ \int _{\tau _n}^{\tau _{n+1}} {{\textbf {X}}}(t) \,\mathrm dt\vert {Z}(\tau _n) = {z}\right] = \frac{{\textbf{x}}({z})}{{\Lambda }_0({z})}, \end{aligned}$$

which is computed in the Appendix A.2 along with the remaining calculations.

#### Remark 3

The expression (13) has a rather astonishing interpretation that can be understood as a consequence of the waiting time paradox (Feller 1971). Suppose the system operates in stationarity. For simplicity, we assume $X$ is one-dimensional. If we choose a random time *t*, then with probability ${\pi }({z})$ we hit an interval $\tau _n \le t < \tau _{n+1}$ with a ’label’ ${Z}(t) = {z}$. Call this event $I_{z}$. The time point adds ${\mathbb {E}}[X(t)\vert I_{z}]$ to the mean if we think of the stationary mean as $\sum _{{z}\in {\mathcal {Z}}} {\pi }({z})\int _{0}^\infty {\mathbb {E}}[X(s)\vert I_{z}] \,\mathrm dF_{z}(s)$, and $F_{z}(s)$ is the distribution of $s = t-\tau _n$ when in ${Z}(t) = {z}.$ Looking at the structure of expression (13), the integral evaluates to $x({z}) = {\mathbb {E}}[X(\tau _{n+1})\vert I_{z}]$. Why is this paradox? We might ad hoc assume that *t* lands, on average, at some centered location within the interval $[\tau _n, \tau _{n+1}]$, in particular it is, on average, smaller than $\tau _{n+1}$. The progression of $s \mapsto {\mathbb {E}}[X(s)\vert I_{z}] = Y(s)$, see Eq. (7), is strictly monotone. Imagine it is increasing (decreasing). Then $t < \tau _{n+1}$ implies ${\mathbb {E}}[X(t)\vert I_{z}] <(>) {\mathbb {E}}[X(\tau _{n+1})\vert I_{z}]$. By the monotonicity of the expectation, this implies $\int _{0}^\infty {\mathbb {E}}[X(s)\vert I_{z}] \,\mathrm dF_{z}(s) <(>) x({z})$. In contrast, Theorem 3 informs us that equality holds. This can be understood as a consequence of the waiting time paradox. A uniformly random time point *t* satisfies that $\tau _{n+1}-t$ is exponentially distributed with parameter ${\Lambda }_0({z})$ by the memory-less property of the exponential distribution. However, due to symmetry reasons in the uniform choice of *t*, the distance $t-\tau _n$ of the last ${Z}$-jump in backwards time is also exponentially distributed with parameter ${\Lambda }_0({z})$. To deviate from the main line of thought, the interval that *t* lands in has, on average, twice the expected length. This paradox is resolved by the size bias effect: longer intervals have a higher chance to be hit by the point *t*. The explicit size-biased distribution of $\tau _{n+1}-\tau _n$ is provided in (Feller 1971). Returning to the main line of thought, a randomly chosen time point is actually an average endpoint, regarded from the perspective of a randomly chosen interval. The difference lies in the random choice of a time point versus the random choice of an interval. The waiting time paradox permits the slim formulation of Theorem 3, once the recursion in Proposition 2 is solved.

Note that for the derivation of ESME to work it is crucial to assume the conditionally linear form of the propensities in Eq. (3). From this assumption we obtained Eq. (7) that are closed in the conditional first moment ${{\textbf {Y}}}(t)$. As a further consequence of the linear form the update function *g* in Eq. (9) is linear in ${{\textbf {Y}}}(\tau _n)$, see Eq. (8), from which we obtain the linear recursions in Eq. (10). If the linear assumption in Eq. (3) was dropped we would not obtain closed equations as in Eq. (7) to begin with. Depending on the functional form of the propensities the equation would involve higher order conditional moments, e.g., for second-order mass-action reactions, or the conditional expectation of rational functions, e.g., for Hill propensities. The analogue to the variable ${{\textbf {Y}}}(t)$ would need to summarize all the dependencies and would be generally of infinite dimension. A conditional moment closure or other projection methods would generally be required to reduce it to finite dimensions. This may result in non-linear *g* in Eq. (9) and in non-linear recursions. One way to obtain a linear equation as in Eq. (7) would be the use of conditional probabilities ${\mathbb {P}}[{{\textbf {X}}}(t) = {\textbf{x}}\vert {Z}_{[0,t]}]$, indexed over all ${\textbf{x}}\in {\mathbb {N}}^d$, together with an appropriate closure scheme. We anticipate that this can become computationally prohibitive. However, it might be feasible for systems that only access finitely many states, e.g., due to conservation relations. For instance, this is the case if every reaction is a conversion reactions, i.e., $\sum _{i=1}^{d}S_{ij} = \sum _{i=1}^{d}P_{ij}$ for all *j*. While the functional form was required to be linear in the state ${\textbf{x}}$, we emphasize that the dependence in the environment component ${z}$ can be of arbitrary functional form.

### Quantification of environmental shares

The stationary mean is a composite result of the subsystem existing in different environmental states. Thus, we aim to quantify the share that each environmental state contributes to the value of the stationary mean. First, let us fix a subsystem species $1 \le i \le d$ and consider its stationary mean ${\mathbb {E}}[X_{i, \infty }]$. When we computed the stationary mean following Eq. (17), we sorted the summands by the environmental states ${Z}(\tau _n) = {z}$. Inspired by Eq. (18), we define the *environmental share* that environmental state ${z}$ contributes to the stationary mean of species *i*, as

21 $$\begin{aligned} \alpha _i({z}):= \Theta _i^{-1}{\mathbb {E}}\left[ \mathbb {1}({Z}(\tau _n) = {z})\int _{\tau _n}^{\tau _{n+1}} X_i(t)\,\mathrm dt \right] \end{aligned}$$

with the normalization

$$\begin{aligned} \Theta _i:= \sum _{{z}\in {\mathcal {Z}}}{\mathbb {E}}\left[ \mathbb {1}({Z}(\tau _n) = {z})\int _{\tau _n}^{\tau _{n+1}} X_i(t)\,\mathrm dt \right] . \end{aligned}$$

We note that, by definition, $\sum _{{z}\in {\mathcal {Z}}} \alpha _i({z}) = 1$. It holds

22 $$\begin{aligned} \alpha _i({z}) = \frac{{\pi }({z})x_i({z})}{{\mathbb {E}}[X_{i, \infty }]}, \end{aligned}$$

because ${\mathbb {E}}[\mathbb {1}({Z}(\tau _n) = {z})\int _{\tau _n}^{\tau _{n+1}} X_i(t)\,\mathrm dt ] \propto {\pi }({z})x_i({z})$ by Eq. (20) and Proposition 1.

## Case studies

We analyze several small chemical reaction networks and gene regulatory motifs, including an example relevant in synthetic biology, to demonstrate how the stationary mean (ESME, Theorem 3) and the environmental shares can provide insight into the properties of the system. Our focus is on comparing the stationary mean of the Q.SS model and ESME to evaluate the effect of the random environment on the mean of the embedded system. ESME requires the generator and the stationary distribution of the Markov environment, as well as the reaction rate constants of the linear subsystem. The generator and reaction rate constants are specified by the model, whereas the stationary distribution can be obtained numerically, or in special cases analytically, e.g., for the class of monomolecular CRNs (Jahnke and Huisinga 2007).

### Birth-death process in a random environment

**Fig. 2:**
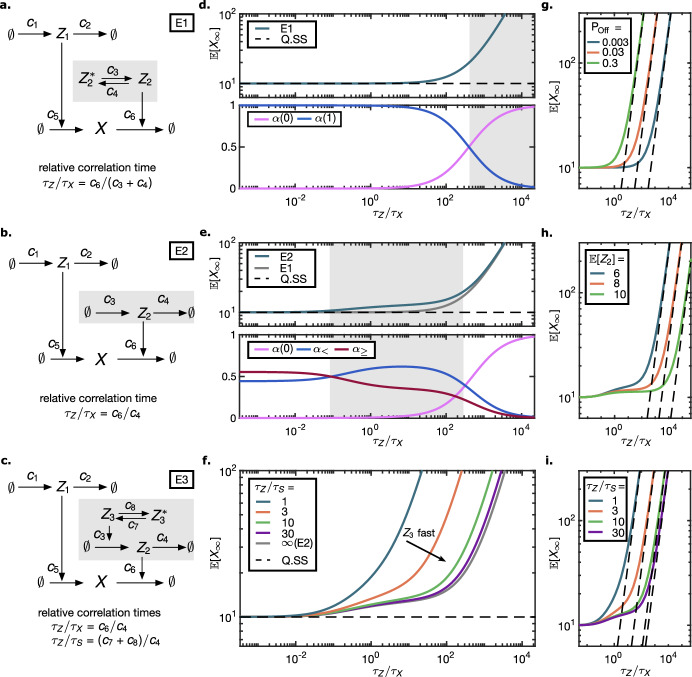
**a–c** Environment schemes for the birth-death process $X$. The two processes $Z_1$ and $Z_2$ modulate the birth and the death rate, respectively. **d** *Upper panel*, **e** *Upper panel*, **f** The Stationary mean as a function of the relative correlation time systematically exceeds the Q.SS mean, to which it converges for fast environment (valid Q.SS assumption). **d** *Lower panel*. The contributions of $Z_2$ to the stationary mean (E1). For slow environment the share of $Z_2= 0$ dominates. **e** *Upper panel*. Three regimes for E2 can be distinguished: the asymptotic, the intermediate and the degenerate regime. The matched plot of E1, Eq. (26), shows agreement in the fast and slow regimes and a discrepancy for the intermediate regime. *Lower panel*. The contributions of $Z_2= z$ for $z \in \{0\}, (0,{\mathbb {E}}[Z_2]), [{\mathbb {E}}[Z_2], \infty )$ to the stationary mean indicate the distinct regimes. **f** Mutable $Z_2$ synthesis (E3): The different curves reflect different relative speeds between environmental components $Z_2, Z_3$. For fast $Z_3$ the muting is neglectable and the environmental effect E2 from **e** is recovered. For slow $Z_3$ the deviation from the Q.SS mean increases. **g, h, i** The asymptotic behaviour for E1–E3 as the relative subsystem speed tends to $\infty$. The log-log plot demonstrates that ${\mathbb {E}}[X_\infty ]$ increases proportional with the relative correlation time. The proportionality constant was computed according to Sect. 4.1.4 and is shown as dashed line. Different values for the **g** percentage of inactivity ${\mathbb {P}}[Z_2= 0]$ (E1), **h** environment mean (E2), **i** relative speed between environmental components (E3) are shown. The relative correlation times $\tau _Z/\tau _X = c_6/(c_3+ c_4)$ (E1), $c_6/c_4$ (E2, E3) progress from slow to fast subsystem (hence slow environment) in increasing direction. All parameter choices kept the Q.SS mean ${\mathbb {E}}[X_\infty ^{\text {Q.SS}}]=10$ and the ratio $c_5/c_6= 1$ fixed. For E2, E3 ${\mathbb {E}}[Z_2] = 8$ was chosen unless indicated otherwise, while in E1 ${\mathbb {E}}[Z_2]$ was calibrated to match ${\mathbb {P}}[Z_2=0]$ with E2. For E3 ${\mathbb {E}}[Z_3] = 0.8$ was held constant when varying $\tau _Z/\tau _S = (c_7+ c_8)/c_4$

First, we illustrate the effect of three different stochastic environments E1–E3 (Fig. 2a–c) on the stationary mean of a birth-death process $X$. The modulated first-order death reaction can be seen as a bimolecular reaction. Here, we demonstrate the effect of different relative speeds between the environment $Z_2$ and the subspecies $X$. The environments E1–E3 modulate the birth and death rates independently. The birth modulation via a birth-death process $Z_1$ is the same in all cases, while the complexity of the death rate modulation $Z_2$ increases.

#### Death modulation via random telegraph (E1)

First, we considered the scenario where the death rate is modulated by a two-state Markov process (Fig. 2a). A two-state modulation highlights the effect that the Off state $Z_2^*$ has on $X$. When the instant decay rate is zero, the molecular numbers of $X$ increase unboundedly. We call these phases excursions. We expect the stationary mean to depend on (i) the length and (ii) the frequency of excursions, because (i) the temporal average during one excursion increases with the length of the excursion, and (ii) if excursions occur more frequently, the excursion average value is weighted more strongly. The frequency can be characterized by $P_\textrm{Off}:= {\mathbb {P}}[Z_2= 0]$, whereas the length of excursions is proportional to the relative correlation time. The autocorrelation function of the random telegraph process, as well as the birth-death process, is of the form $e^{-t/\tau }$, where *t* is the time lag and $\tau$ is the correlation time. Thus, for the random telegraph process $Z_2$ with On and Off switching rate $c_3, c_4$, the correlation time is $\tau _Z = (c_3+ c_4)^{-1}$, while for the birth-death process with constant degradation $c_6$ it is $\tau _X = c_6^{-1}$. With these definitions, the expression of the stationary mean (derived using ESME, Eq. (15), see Appendix B.1) takes the form

23 $$\begin{aligned} {\mathbb {E}}[X_\infty ] = {\mathbb {E}}[X_\infty ^\mathrm {Q.SS}]\left( 1 + P_{\textrm{Off}}\frac{\tau _Z}{\tau _X}\right) . \end{aligned}$$

In the queuing literature, Baykal-Gursoy and Xiao (2004) already derived an equivalent expression in terms of rate constants, along with other characteristics for the model, e.g., stationary distribution, using a generating function approach. The bracket term is larger than 1, yielding a systematic positive deviation compared to the Q.SS as a reference model (Sect. 2.4). Note that this can be expected from the moment equations. The mean equation for $X$ is

24 $$\begin{aligned} \frac{\,\mathrm d}{\,\mathrm dt}{\mathbb {E}}[X(t)]&= c_5{\mathbb {E}}[Z_1(t)] - c_6{\mathbb {E}}[Z_2(t)X(t)] \end{aligned}$$

25 $$\begin{aligned}&= c_5{\mathbb {E}}[Z_1(t)] - c_6{\mathbb {E}}[Z_2(t)]{\mathbb {E}}[X(t)] - c_6{{\,\textrm{Cov}\,}}[Z_2(t), X(t)]. \end{aligned}$$

The first two terms in Eq. (25) would yield the Q.SS dynamics. Since $Z_2$ and $X$ are negatively correlated, the stationary mean is larger compared to the Q.SS mean. As Eq. (23) shows, the deviation is proportional to the separation of time scales $\tau _Z/\tau _X$ and to the probability ${\mathbb {P}}[Z_2= 0]$.

Figure 2d (upper panel) portrays the stationary mean ${\mathbb {E}}[X_\infty ]$ as a function of the relative correlation time $\tau _Z/\tau _X$, given that the Q.SS mean and $P_{\textrm{Off}}$ are constant. Increasing the relative correlation time can be achieved by either accelerating $X$ or decelerating $Z_2$. Figure 2d (lower panel) shows how the share of $Z_2= 0$ increases with the mean waiting time in $Z_2= 0$ (or decreases with increasing speed of $Z_2$). In the asymptotic regime $\tau _Z/\tau _X \rightarrow 0$, the stationary mean reaches the Q.SS mean, confirming that the Q.SS assumption is valid for sufficiently fast $Z_2$ or, equivalently, slow $X$.

#### Death modulation via birth-death process (E2)

We next investigated whether the generic expression Eq. (23) still holds when the death modulator $Z_2$ is itself a birth-death process (Fig. 2b). To this end, we altered $\tau _Z = c_4^{-1}$ and $P_\textrm{Off} = \exp (-{\mathbb {E}}[Z_2])$ and asked whether

26 $$\begin{aligned} f\left( \frac{c_6}{c_4}\right) = {\mathbb {E}}[X_\infty ^{\mathrm {Q.SS}}] \left( 1 + \frac{c_6}{c_4}\exp (-{\mathbb {E}}[Z_2])\right) \end{aligned}$$

approximates ${\mathbb {E}}[X_\infty ]$, computed via ESME in Appendix B.2. The approximation is valid for the extreme cases of (i) $c_4$ large compared to $c_6$ or (ii) $c_4$ small compared to $c_6$ (Fig. 2e, upper panel). Namely, we found ${\mathbb {E}}[X_\infty ] = {\mathcal {O}}(f(\frac{c_6}{c_4}))$ for (i) $\frac{c_6}{c_4} \rightarrow \infty$ and (ii) $\frac{c_6}{c_4} \rightarrow 0$. The means ${\mathbb {E}}[Z_1]$ and ${\mathbb {E}}[Z_2]$, as well as $c_5/c_6$, were fixed.

Second, we partitioned the states of $Z_2$ into three classes: the zero state, the non-zero states below the mean, and the states equal to or above the mean. The relative speed $\tau _Z/\tau _X$ of the environment defines which of these three classes dominates in terms of the corresponding environmental share, i.e., the effect on the stationary mean. In order to interpret the deviation of E2 from E1, we quantified environmental shares according to the three classes of environment states:

$$\begin{aligned} \alpha (0), \quad \alpha _{<{{\bar{{z}}}}}:= \sum _{k = 1}^{\lceil {\mathbb {E}}[Z_2] - 1 \rceil }\alpha (k), \quad \alpha _{\ge {{\bar{{z}}}}}:= \sum _{k = \lceil {\mathbb {E}}[Z_2] \rceil }^{\infty }\alpha (k). \end{aligned}$$

We found that, depending on the relative speed of the environment, the subsystem can be in one of three phases (Fig. 2e, lower panel). For a small $c_6/c_4$ ratio, the non-zero shares $\alpha _{<{\bar{{z}}}}$ and $\alpha _{\ge {\bar{{z}}}}$ both contribute significantly, with $\alpha _{\ge {\bar{{z}}}}$ showing a slight dominance over $\alpha _{<{\bar{{z}}}}$, while the share of $\alpha (0)$ is negligible. For a medium $c_6/c_4$ ratio, $\alpha _{<{\bar{{z}}}}$ takes the lead in dominance, while $\alpha (0)$ is still negligible. Finally, for large $c_6/c_4$, the share $\alpha (0)$ dominates the contribution to the mean. Returning to the upper panel of the Fig. 2e, we confirm that the mean ${\mathbb {E}}[X_\infty ]$ as a function of $\tau _Z/\tau _X$ undergoes the same phase transitions.

The qualitative behavior for large $\tau _Z/\tau _X$ is driven by unbounded excursions in the state $Z_2=0$. However, in biological systems, these are generally prevented by, e.g., a leakage in the death rate. Upon introducing a base death rate $\lambda _0$, we expect the stationary mean to saturate at the upper bound as the relative environmental speed approaches zero. To demonstrate this, we generalize the propensity of the death reaction to ${f}(x \vert z) = (c_6z + \lambda _0)x$.  The Fig. 5 indicates four qualitatively different regimes of the stationary mean over the relative correlation time. In particular, the three phases of the model E2 analysis (Fig. 2e) persist, whereas the fourth phase with the largest $\tau _Z/\tau _X$ reaches saturation.

#### Mutable synthesis of the modulator (E3)

We next considered the case where the modulating process $Z_2$ is itself modulated by another process, $Z_3$ (Fig. 2c). The modulator $Z_3\leftrightarrow Z_3^*$ is a two-state Markov process that acts as a switch with On ($Z_3$) and Off ($Z_3^*$) states, switching rates $c_7, c_8$, and correlation time $\tau _S = (c_7+ c_8)^{-1}$. Here, $Z_2$ can be seen as a regulatory protein produced from a promoter $Z_3$ that alternates between On and Off states. Then $Z_2$ is a Markov-modulated birth-death process and, as such, represents an example of a non-Markovian death rate modulator. Since the joint environment $(Z_2, Z_3)$ is Markovian, ESME applies (see Appendix B.3).

We varied the relative correlation time $\tau _Z/\tau _X = \frac{c_6}{c_4}$ while keeping the ratios $\frac{c_7}{c_4}$, $\frac{c_8}{c_4}$, and $\frac{c_3}{c_4}$ constant. Furthermore, we varied the relative speed of the environmental components by additionally varying the relative correlation time $\tau _Z/\tau _S=\frac{c_7+ c_8}{c_4}$ while keeping the fraction of time the modulator is active, ${\mathbb {E}}[Z_3]= \frac{c_7}{c_7+ c_8}$, constant. For a large relative speed $\frac{c_7+ c_8}{c_4}$, the original model with a constant birth rate $c_3{\mathbb {E}}[Z_3]$ is recovered as expected.

Comparing the stationary means at different relative switching speeds of the modulator (Fig. 2f), we found the following. For slower relative speeds, the deviation from the Q.SS mean becomes more pronounced already at smaller correlation times $c_4^{-1}$, and the intermediate phase vanishes. Meanwhile, Fig. 6a visualizes how the entry into the degenerate regime depends on $\tau _Z/\tau _S$ and ${\mathbb {E}}[Z_3]$. The muting prolongs the excursions of $X$ in the zero or sub-average $Z_2$ states.

#### Stationary mean for slow environment

We considered each of the environments E1–E3 without leakage in the death rate of $X$ and analyzed the behaviour for a slow environment and a fast subsystem, i.e., $\tau _Z/\tau _X \rightarrow \infty$. We aimed at isolating the effect of the time scale separation. For this purpose, we kept the means and the relative speed of the environment components, as well as $c_5/c_6$, fixed. As the plots of the environmental shares (Figs. 2d, e, 6b) suggest, the state $Z_2= 0$ is the only one that contributes in the limit case $\tau _Z/\tau _X \rightarrow \infty$. From this, we derived

27 $$\begin{aligned} {\mathbb {E}}[X_\infty ] = {\mathcal {O}}\left( \frac{\tau _Z}{\tau _X}\right) , \end{aligned}$$

see Appendix B.4, i.e., the stationary mean grows proportionally to the time scale differences. The parallel asymptotes in the log-log plots with unit slope in the Fig. 2g–i reflect this dependence.

#### Application to a division-dilution model

In a model similar to E1, Sect. 4.1.1, Beentjes et al. (2020) studied the dilution of protein copy numbers due to cell division. The mean copy number in a single lineage increased in a model with Erlang distributed division time and binomial partitioning at cell division (model III in the reference) compared to the same model with deterministic division times (model II) and to a birth-death model with averaged effective degradation rate (model I). We can attribute this deviation to the random cell division times. To demonstrate this attribution, we elaborate on the analogy to the random duration of excursions in E1. Let the extrinsic noise component (the environment) be the cell cycle duration. To formalize this, we introduce the time points of division, denoted by a sequence of random variables $0 =: \tau _0< \tau _1< \tau _2 < \dots$, such that the $\tau _{i+1} - \tau _i$ are i.i.d. Let *X*(*t*) be the number of proteins that increases on average with rate $\lambda$ between environment jumps, be it by geometrically distributed bursts or by simple birth reactions. Furthermore, let $X(\tau _i)$ follow a Binomial distribution with $p = 1/2$ and $N = X(\tau _i -)$. If the protein production, the binomial partition at division, and the environment jumps are all assumed to be stochastically independent, it is easy to see that $Y(t):= E[X(t)\vert (\tau _i)_i]$ evolves as $\dot{Y}(t) = \lambda$ between the jumps and is set to $Y(\tau _i) = 1/2 \cdot Y(\tau _i-)$ at the environment jumps. It is furthermore easy to derive ${\mathbb {E}}[Y(\tau _i)] = \lambda {\mathbb {E}}[\tau _1]$ for the stationary system. Then, analogous to the derivation in Sect. 3.3, we obtain

$$\begin{aligned} {\mathbb {E}}[X_\infty ]&= \lim _{N \rightarrow \infty }\frac{\frac{1}{N} \sum _{i = 1}^N \int _{\tau _{i-1}}^{\tau _{i}} Y(t) \,\mathrm dt}{\frac{1}{N} \sum _{i = 1}^N \tau _i - \tau _{i-1}} = \lambda {\mathbb {E}}[\tau _1] + \frac{1/2 \lambda {\mathbb {E}}[\tau _1^2]}{{\mathbb {E}}[\tau _1]} \\&= \frac{\lambda }{2} \left( 3{\mathbb {E}}[\tau _1] + \frac{{\mathbb {V}}\textrm{ar}[\tau _1]}{{\mathbb {E}}[\tau _1]}\right) . \end{aligned}$$

For (*N*, *N*/*y*)-Erlang distributed $\tau _1$, i.e., ${\mathbb {E}}[\tau _1] = y$ and ${\mathbb {V}}\textrm{ar}[\tau _1] = y^2/N$, this is in accordance with Eq. (39) in Beentjes et al. (2020), which was derived using a generating function approach. Compared with our two-state Markov model of alternating linear increase and exponential decay in *X*, the division-dilution model replaces the periods of exponential decay by instantaneous decays at divisions. In both models, the stochasticity in the duration of excursions (referred to as cell cycle length variability in the reference) causes an increase in the stationary mean. While in the reference paper this is the single cause of the increase compared to the model I, in our case the increase is caused by an interplay of the random duration, the frequency of the excursions, and the time scale separation between *Z* and *X*. Note that the reference models (model I and Q.SS, respectively) are constructed differently. The degradation rate of the model I was tuned such that the stationary mean matches with that of the deterministic division-dilution model II. As a consequence, it encodes the time scale of the environment, whereas in the Q.SS reference model, the degradation rate is uncoupled from the environment time scale. This uncoupling provides a degree of freedom which causes a larger relative increase of the stationary mean value in our case study than in Beentjes et al., compared to the respective reference models.

### Synthetic controller mitigating the heterogeneous degradation rate

**Fig. 3:**
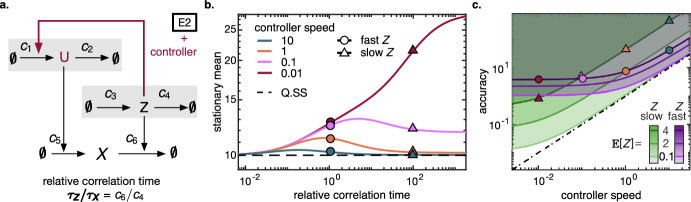
**a** Reaction scheme for the environment with controller. The birth-death environment E2 is mitigated by a controller that senses the environment and regulates the birth rate of $X$ accordingly. **b** The stationary mean as a function of the relative correlation time $\tau _Z/\tau _X$. Different relative controller speeds $c_2/c_6$ are plotted. Circles (triangles) indicate fast (slow) environment. **c** Accuracy $\Delta ^{-1}$ as a function of controller speed $c_2/c_6$ for different values of environment mean ${\mathbb {E}}[Z]$ and speed $c_4/c_6$. Green (purple) indicates slow (fast) environment. The mean value ${\mathbb {E}}[Z] = 4$ is highlighted by triangles (slow) or circles (fast) to match **b**. The slow environment achieves lower accuracy than the fast one for inactive controller (controller speed near 0). In order to improve accuracy, less increase in controller speed is needed for slow than for fast environment. As a consequence the accuracy curves for slow and fast environment intersect. The dashed line indicates the the critical controller speed needed to reach a given accuracy level for all environment mean and speed. The slow and low environment exhausts this universal accuracy-controller relation. Parameters were ${\mathbb {E}}[X_\infty ^{\mathrm {Q.SS}}] = c_1/c_2= 10, c_6{\mathbb {E}}[Z_2] = 4 (2, 0.1), c_6/c_4= 1$ (circles, purple), 100 (triangles, green) (color figure online)

One goal of synthetic biology is to design circuits that are robust to environmental changes. The setpoint objective specifies a target copy number or concentration at which the species of interest should be kept robustly, i.e., the concentration is supposed to re-adapt when environmental changes perturb it. We considered the setting in which an environmental birth-death process modulates the degradation of $X$ (Fig. 3a). A controller species senses the environment and acts on the birth rate of $X$ to attenuate the effect and achieve the setpoint objective for $X$ (Zechner et al. 2016, Fig S.9c). We chose the Q.SS mean as the setpoint, and as the deviation measure we employed the relative deviation

28 $$\begin{aligned} \Delta = \frac{{\mathbb {E}}[X_\infty ] - {\mathbb {E}}[X^\mathrm {Q.SS}_\infty ]}{{\mathbb {E}}[X^\mathrm {Q.SS}_\infty ]}, \end{aligned}$$

or the accuracy measure $\Delta ^{-1}$, respectively. In the previous sections we saw that $Z= 0$ can be the main driver of deviations from the Q.SS. The controller $U$ works against this effect: during phases of otherwise unbounded $X$ excursions, the birth rate of $X$ is now down-regulated by $U$ and, in the extreme case, comes to a halt at a plateau (for details see Appendix B.5).

With stochastically independent birth and death modulation that we considered so far, the stationary mean was not affected by fluctuations in the birth modulation, and we could apply the Q.SS assumption on $Z_1$. Here, on the contrary, the time scale of the controller species $U$ matters in reacting robustly to the environment. Figure 3b depicts the stationary mean of $X$ for different controller speeds $c_2/c_6$. The deviation $\Delta$ gets more pronounced for a slower controller, whereas a fast controller achieves better accuracy $\Delta ^{-1}$. For the effect to become apparent, the environment needs to be sufficiently slow ($\tau _Z/\tau _X$ large). When the controller operates slowly, it does not have the attenuating effect. In this regime, the target species achieves a base accuracy that depends on a given environment speed and its mean (see Fig. 3c). The base accuracy decreases when the environment gets lower in mean or slower in time scale. In particular, a slow controller achieves worse accuracy in a slow environment compared to a fast one. As a contrary effect, as the controller speeds up, it departs to a better accuracy later in a fast environment than in a slow one. That is why in Fig. 3c the accuracy curves for slow and fast environment intersect, which also explains the local maxima in Fig. 3b. The slower environment - although having a lower base accuracy as a handicap - can be compensated for (in terms of accuracy) already at a slower controller speed.

As a key question, we asked at which time scale the controller must operate to mitigate environmental perturbations with a given accuracy. To formalize this, we request the deviation in Eq. (28) to stay below a critical margin $\Delta ^*$ or the accuracy to stay above $1/\Delta ^*$. Interestingly, we found that for each accuracy margin, a critical controller speed can be chosen that operates universally for all environment speeds and means (see the dashed line in Fig. 3c). For fixed $c_5/c_6$ and $c_1/c_2$, the critical relative controller speed is given by

29 $$\begin{aligned} c_2^*/c_6= (\Delta ^*)^{-1}. \end{aligned}$$

When the environment changes in mean or time scale, the controller holds the accuracy within the tolerated margin. Since the robustness that we analyze is defined via the steady state behaviour, our statement is restricted to environmental changes that occur so rarely that the steady state can be reached between changes (see Appendix Fig. 8).

Which environment speed and mean exhaust the critical accuracy? It is not the fast and furious (i.e., large mean) environment that causes deviation from Q.SS. For fast environment, the degradation rate of $X$ averages out to the mean, making the Q.SS assumption valid. For large (furious) environment, the main driver of deviation 0 is hardly visited. In addition, the furious environment boils up the controller to act in a regime where it has a higher signal-to-noise ratio. This leaves the slow and low $Z$ to exhaust the critical deviation. During excursions $(Z= 0)$, the average dynamics follows

$$\begin{aligned} \dot{u}&= -c_2u, \quad u(0) = {\mathbb {E}}[U\vert Z= 1] = c_1/c_2\\ \dot{x}&= c_5u, \quad x(0) = {\mathbb {E}}[X^\mathrm {Q.SS}_\infty ]. \end{aligned}$$

Consequently, the deviation for an infinitely long excursion rises, on average, to the value

$$\begin{aligned} x(\infty ) - {\mathbb {E}}[X^\mathrm {Q.SS}_\infty ]= \frac{c_5c_1}{c_2} \int _0^\infty e^{-c_2t}\,\mathrm dt = \frac{{\mathbb {E}}[X^\mathrm {Q.SS}_\infty ]c_6}{c_2}. \end{aligned}$$

The excursion becomes the single dominating share as ${\mathbb {E}}[Z]$ tends to 0, and $\tau _Z\rightarrow \infty$ justifies infinitely long excursions. Since this dominating case is linear, the average dynamics is justified and the heuristic derivation of Eq. (29) can be made rigorous.

In summary, we observed that the accuracy margin was exhausted by the regime of slow and low environment, leaving the controller with the simple task to react as fast as to guarantee that a plateau is reached within the tolerated deviation, on average. This finding joins the variations on the theme ’faster sensor molecules achieve higher accuracy’. Note that the setting and the control objective differ from Lestas et al. (2010). In the latter, a sensor molecule recorded the progression of the target species, while here it senses the environment. The objective of suppressing fluctuations, i.e., the variance, in the controlled species induced the quartic root law on the sensing event counts. Here, in contrast, we asked for the stationary mean to stay within a tolerance of the setpoint, and this induced an inverse proportional law on the speed with which the controller responds to the environment. To summarize, both findings show that, when under the influence of a random environment, the accuracy can be increased at the cost of a faster sensor molecule. Our finding stands out due to its independence of size and speed of $Z$ and the proportional relation in Eq. (29).

### Stochastic toggle switch in a random environment

In the previous examples, we observed the effects of the modulation of the stochastic birth-death process given different architectures of this process. The next question of interest is whether the modulation effects propagate into the networks constructed from several such processes that interact with each other. Here, we consider a simple genetic toggle switch without cooperative binding, which is one of the best-studied small gene networks that induces bistability (Lipshtat et al. 2006). A balance of a toggle switch is known to be sensitive to noise (Tian and Burrage 2006), and we hypothesise that the effect of the noise in the stochastic environment should be visible at the level of the mean expression of the component genes.

**Fig. 4:**
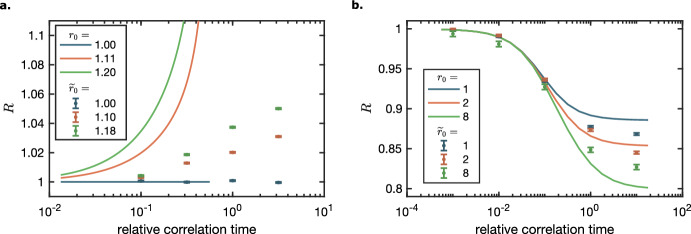
The relative asymmetry change ratio $R$ and its counterpart ${\widetilde{R}}$ as a function of the relative correlation time $\tau _Z/\tau _X$ for different baseline asymmetry $r_0$ and ${\widetilde{r}}_0$ values, computed for the subsystem species of **a** toggle switch with fixed parameters $E[X^\mathrm {Q.SS}_{1,\infty }] = 30$, ${\mathbb {E}}[X^\mathrm {Q.SS}_{2,\infty }] = 30, 27, 25$, $b_1= b_2= 0.02$, $c_{6}= 0.01$, with $a_1, a_2$ matched accordingly; and **b** oscillator with fixed parameters $E[X^\mathrm {Q.SS}_{1,\infty }] = 40$, ${\mathbb {E}}[X^\mathrm {Q.SS}_{2,\infty }] = 40, 20, 5$, $b_1= 0.02$, $c_{6}= 0.01$, with $a_1$ and $b_2$ matched accordingly. The fixed parameters of the environment of both toggle switch and oscillator are: $c_1= 300$, $c_2= 100$, ${\mathbb {E}}[Z_2] = 10$. All simulations were performed for $10^6$ time points

**Fig. 5:**
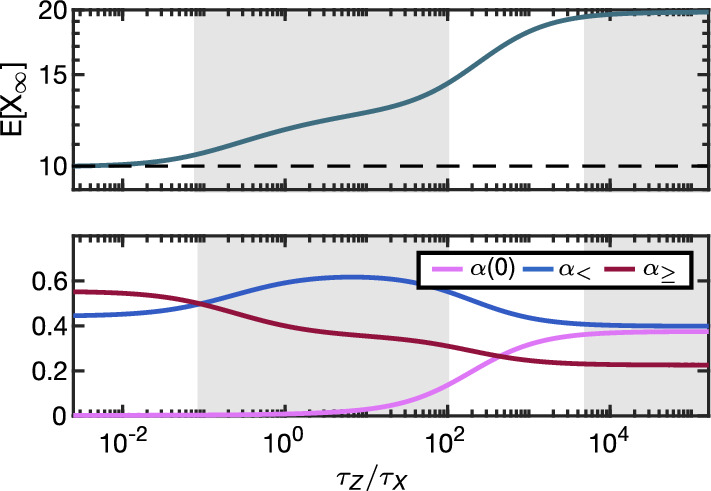
Model E2 with leakage. Upper panel. Stationary mean as a function of $\ln (c_4)$. The stationary mean systematically exceeds the Q.SS mean $\frac{c_1c_5}{c_2c_6(c_3/c_4+ \lambda _0)}=9.97$. Four regimes can be distinguished: the asymptotic regime, the intermediate regime, the rising degenerate regime and the saturating degenerate regime. Lower panel. The contributions of $Z_2$ to the stationary mean. Parameters were $\frac{c_1c_5}{c_2} = 60, \frac{c_3}{c_4} = 6, c_6= 1, \lambda _0= 0.02$

**Fig. 6:**
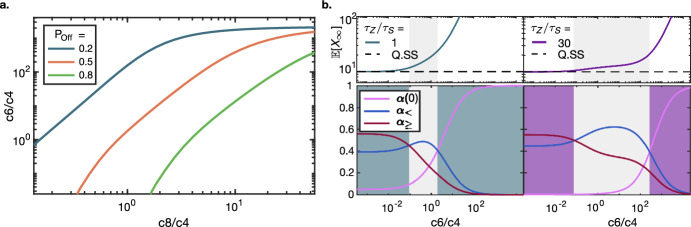
Model E3 mutable $c_3$ synthesis. **a** The figure shows the $c_4$ value at which $\alpha (0)$ and $\alpha _{<{\bar{{z}}}}$ intersect as a function of $\frac{c_8}{c_4}$. Dominant $\alpha (0) > \alpha _{<{\bar{{z}}}}$ indicates that the stationary mean has entered the degenerate regime. The three curves illustrate different values of ${\mathbb {P}}[Z_3= 0]$. For ${\mathbb {P}}[Z_3= 0] = 0.5, 0.8$ and slow enough relative speed $c_8/c_4$ no intersection was found, because $\alpha (0)$ dominated $\alpha _{<{\bar{{z}}}}$ for all $c_4$. The mean ${\mathbb {E}}[Z_2] = 8$ was fixed and $c_3/c_4$ adapted accordingly. Parameters were $c_1= 0.4, c_2= 0.01, c_5= 1, c_6= 0.5$ and ${\mathbb {E}}[X_\infty ^{\mathrm {Q.SS}}] = 10$. **b** The left upper panel shows the slow switching $Z_3$, whereas the right panel shows the fast switching $Z_3$, compare Fig. 2f. The corresponding shares are depicted in the lower panels. Parameters were as in Fig. 2f

We model the genetic toggle switch as a subsystem of two processes that are modulated by a shared environment $(Z_1, Z_2)$ in the same way as the single gene is modulated in the Sect. 4.1.2. These processes interact with each other by reducing the birth rate of their counterparts (Fig. 4a). In terms of a gene network, each process here is the number of proteins expressed by a gene. Their birth and death rates are modulated by the environment, and the birth rates are decreased in the presence of proteins expressed by the other gene. This model can be described by the following set of stochastic reactions:

$$\begin{aligned} \begin{matrix} {{\,\textrm{R}\,}}_1, {{\,\textrm{R}\,}}_2&{}: \qquad \emptyset \overset{c_1}{\longrightarrow }Z_1\overset{c_2}{\longrightarrow }\emptyset &{}\\ {{\,\textrm{R}\,}}_3, {{\,\textrm{R}\,}}_4&{}: \qquad \emptyset \overset{c_3}{\longrightarrow }Z_2\overset{c_4}{\longrightarrow }\emptyset &{}\\ {{\,\textrm{R}\,}}_5&{}: \qquad Z_1\overset{g_1}{\longrightarrow }Z_1+X_1,&{}g_1= a_1-b_1X_2\\ {{\,\textrm{R}\,}}_6&{}: \qquad Z_1\overset{g_2}{\longrightarrow }Z_1+X_2,&{}g_2= a_2-b_2X_1\\ {{\,\textrm{R}\,}}_7&{}:\qquad X_1+Z_2\overset{c_{6}}{\longrightarrow }Z_2&{}\\ {{\,\textrm{R}\,}}_8&{}:\qquad X_2+Z_2\overset{c_{6}}{\longrightarrow }Z_2&{}\\ \end{matrix} \end{aligned}$$

Reactions ${{\,\textrm{R}\,}}_1,{{\,\textrm{R}\,}}_2,{{\,\textrm{R}\,}}_3,{{\,\textrm{R}\,}}_4$ model the stochastic environment. Reactions ${{\,\textrm{R}\,}}_5,{{\,\textrm{R}\,}}_6$ describe gene expression and regulation modulated by the stochastic environment $Z_1$. Rates $a_1,a_2$ correspond to the rate of expression of a the fully active gene, whereas coefficients $b_1,b_2$ are proportional to the strength of repression of the corresponding regulatory molecules produced by the counterpart gene. Finally, reactions ${{\,\textrm{R}\,}}_7,{{\,\textrm{R}\,}}_8$ describe the decay of the gene products modulated by the stochastic environment $Z_2$. The subsystem species $X_1$ and $X_2$, besides interacting directly, are additionally coupled by the confounding factors $Z_1$ and $Z_2$.

Note that here we use a linear function to model repression, instead of, e.g., a Hill function, which would be a standard choice for this purpose (Santillán 2008; Zhu et al. 2007). This decision is based on the fact that a standard repression model employs a non-linear reaction propensity, whereas ESME can work only with linear subsystems. In order to adhere to an implicit assumption that numbers of each species of the subsystem cannot be negative, we only considered the region of the parameter space of the model where the following is true. First, no share contributed by any state of the environment can be negative. Second, the total share contributed by the states that stabilize at negative values of the subsystem species cannot exceed 0.01%, which keeps its impact on the modulated mean expectation negligibly small (see also Appendix C.1).

To investigate the effect of the environmental modulation on the asymmetry of the toggle switch in the unimodal regime, we first define the asymmetry of the switch as a ratio of the stationary means of its component processes:

$$\begin{aligned} r= \frac{{\mathbb {E}}[X_{1,\infty }]}{{\mathbb {E}}[X_{2,\infty }]}. \end{aligned}$$

In the case of a constant environment, we denote this ratio as $r_0$, the baseline asymmetry of the switch:

$$\begin{aligned} r_0= \frac{{\mathbb {E}}[X^\mathrm {Q.SS}_{1,\infty }]}{{\mathbb {E}}[X^\mathrm {Q.SS}_{2,\infty }]}. \end{aligned}$$

Then we can quantify the effect of a given environmental modulation on this asymmetry by computing the relative asymmetry change ratio $R= \frac{r}{r_0}$.

Here, we chose the model parameters that result in three different $r_0$ ratios and, for each of these cases, varied the relative correlation time $\tau _Z/\tau _X = \frac{c_6}{c_4}$ while keeping the ratio $\frac{c_3}{c_4}$ fixed. The stationary means for various $\tau _Z/\tau _X$ were computed with ESME (Appendix B.6). From Fig. 4a (solid lines), (i) a symmetric switch stays symmetric also in a random environment, (ii) the relative asymmetry in an originally asymmetric switch increases with the relative correlation time, (iii) this effect of the environmental speed on the asymmetry is higher when the baseline asymmetry, $r_0$, is higher. We also note that, for asymmetric switches, the value of $R$ grows towards a vertical asymptote. This asymptote signifies the $\tau _Z/\tau _X$ value at which the weaker gene of the toggle switch (in our example, it is $X_2$) becomes repressed so strongly that its mean approaches 0. The switch model with the repression implemented using linear propensities is descriptive of the modelled system only on the left side of this asymptote (for limitations, see Appendix C.1).

Given that the asymptote is an artifact of the linearization in reactions ${{\,\textrm{R}\,}}_5,{{\,\textrm{R}\,}}_6$, we verified that, apart from it, the observed monotonic dependencies (i), (ii), (iii) of the relative switch asymmetry are indeed the property of the toggle switch in a random environment. For this, we replaced the ${{\,\textrm{R}\,}}_5$ and ${{\,\textrm{R}\,}}_6$ with reactions ${{\,\textrm{R}\,}}_5^*$ and ${{\,\textrm{R}\,}}_6^*$

$$\begin{aligned} \begin{matrix} {{\,\textrm{R}\,}}_5^*&{}:&{} \qquad Z_1\overset{{\tilde{g}}_1}{\longrightarrow }Z_1+X_1,&{}{\tilde{g}}_1= k_1\frac{K_{A_1}}{K_{A_1}+X_2}\\ {{\,\textrm{R}\,}}_6^*&{}:&{} \qquad Z_1\overset{{\tilde{g}}_2}{\longrightarrow }Z_1+X_2,&{}{\tilde{g}}_2= k_2\frac{K_{A_2}}{K_{A_2}+X_1}\\ \end{matrix} \end{aligned}$$

that utilize a Hill function (without cooperative binding), which is a standard way to describe the expression of a gene subject to repression (Santillán 2008; Zhu et al. 2007). Since the exact stationary mean is computationally unfeasible to obtain, we performed stochastic simulations (Doob 1945; Gillespie 1976) of the changed model. To ensure that the models match, we performed a “reverse linearization” between the linear and the Hill function repression models (Appendix B.7). Furthermore, we chose parameters of the reactions ${{\,\textrm{R}\,}}_5^*,{{\,\textrm{R}\,}}_6^*$ such that $r_0$ and ${\widetilde{r}}_0$ take similar values for convenient comparison.

From Fig. 4a, we see that all the three observations (i), (ii), (iii) made with regards to how $\tau _Z/\tau _X$ affects the asymmetry of a toggle switch hold for the model with a nonlinear repression function. However, while the behaviour is the same in terms of the symmetric case (i) and the monotonic properties (ii), (iii), there is a shift along the x-axis between the $R$ and ${\widetilde{R}}$ values. This is likely because the Hill function represses less strongly than its linearization. For the linearized repression, the dominant stable state with high $X_1$ and low $X_2$ is induced at a lower perturbation, i.e., at lower correlation, even driving the $X_2$ to full extinction. We conjecture that an asymptote also occurs for the Hill model, caused by much higher $X_1$ values than $X_2$ values due to excursions, likely also driving $X_2$ to full extinction.

Our method is limited in that it has only access to the means and requires linearization. Thus, we focused on the amplification of asymmetry in the unimodal regime. However, other studies looked into the effect of degradation modulation on trajectory properties, i.e., stability and switching, and on the shape of the distribution, i.e., bimodality. Xu et al. (2014) examined an ODE model of the toggle switch with noise in the degradation rates and found noise induced switching. Holehouse et al. (2020) considered an autoregulatory gene expression system that can exhibit bimodality and showed that bimodality can be induced by noise in the degradation rate, even if the system without noise is in the unimodal regime.

### Stochastic oscillator in a random environment

While some effects of the random environment on the mean levels of the toggle switch components were to be expected, it might seem counter-intuitive that an effect will also be visible in a network with a periodic behavior. In particular, one might object that, in an oscillatory system, the information about the network period is lost when we move from the time series to the mean levels in the analysis. However, an oscillator is also characterized by an amplitude of its components, and we expect that the effects of the random environment on these amplitudes will be visible in the mean levels of the subsystem components. Further, changes in the dynamics of skipped oscillations, which are possible in stochastic oscillators, could propagate into changes in the mean species levels.

Reactions of the oscillator model differ from the reactions of the switch model (Sect. 4.3) only in reaction ${{\,\textrm{R}\,}}_6$, which now models induction of the species $X_2$ by the species $X_1$ with the induction strength coefficient $b_2$ and takes form:

$$\begin{aligned} \begin{matrix} {{\,\textrm{R}\,}}_6&{}:&{} \qquad Z_1\overset{g_2}{\longrightarrow }Z_1+X_2,&{}g_2= b_2X_1,\\ \end{matrix} \end{aligned}$$

Note that here, as in Sect. 4.3, we model repression propensity with a linear function. Thus, we investigated the model only in the subset of its parameter space where no share contributed by any state of the environment is negative and the mean values of the subsystem species stabilize at non-negative values in all considered states of the environment.

We quantified the effect of a given environmental modulation on this oscillator by computing the relative asymmetry change ratio $R= \frac{r}{r_0}$, as in Sect. 4.3, in three different $r_0$ ratios over the varied relative correlation time $\tau _Z/\tau _X = \frac{c_6}{c_4}$ while keeping the ratio $\frac{c_3}{c_4}$ fixed. The stationary means for various $\tau _Z/\tau _X$ were computed with ESME (Appendix B.7). From Fig. 4b (solid lines), $R$ decreases with the relative correlation time for all $r_0$ values, and this effect is more pronounced at higher $r_0$ values. In terms of $X_1$ and $X_2$, this means that a random environment causes ${\mathbb {E}}[X_{1,\infty }]$ to decrease relative to ${\mathbb {E}}[X_{2,\infty }]$, and this effect is stronger when the oscillator has higher ${\mathbb {E}}[X^\mathrm {Q.SS}_{1,\infty }]$ relative to ${\mathbb {E}}[X^\mathrm {Q.SS}_{2,\infty }]$.

As in Sect. 4.3, we verified that the observed effects are the property of the studied network in a random environment and do not qualitatively depend on the linearization assumption made when modelling repression in reaction ${{\,\textrm{R}\,}}_5$ by performing stochastic simulations of the oscillator model with ${{\,\textrm{R}\,}}_5$ replaced by ${{\,\textrm{R}\,}}_5^*$. From Fig. 4b (dots), the results of the simulations are qualitatively the same as those produced by ESME.

## Discussion

In this work, we provided an exact stationary mean evaluation for linear chemical reaction networks in Markov environment, as a way to address (i) the difficulty of deriving the means in networks with bimolecular reactions and (ii) the computational infeasiblity of co-simulating the environment with the subsystem. Namely, the computation of stationary means in chemical reaction networks commonly relies on Monte Carlo simulation, moment closure or the approximation via a linear program with convex constraints. These approaches are often time-consuming or prone to approximation errors. Our expression computes the stationary mean exactly for a particular class of chemical reaction networks, i.e., the ones that can be decomposed into a linear subsystem and an environment, where the modulation is only allowed unidirectionally from environment to subsystem. Often such decomposition does not exist, because the requirements of linearity and unidirectional modulation are limiting. Common violations are the following: (1) the existence of a reaction $A + B \rightarrow C$ where at least one of the three species is in the subsystem, e.g., the MAPK/ERK pathway (Purutçuoǧlu and Wit 2006). If *A* is an environmental species, it needs to be preserved during the reaction, and *A* and *B* cannot be both in the subsystem due to linearity. (2) Systems for which any partition into two sets of species comprises bimolecular reactions in both directions are not feasible. Finally, (3) reaction rates that have a Hill or other non-linear dependency violate the linearity constraint. We suggest that, for non-linear propensities, Monte Carlo simulations with the method of conditional moments (Hasenauer et al. 2014) are used to obtain numerical estimates of the stationary mean. Additionally, our expressions require the stationary distribution of the environment, which further limits our approach.

In the presented case studies, we focused on the effect of a modulated degradation. As a general effect, we found that, as the environment slows down, the mean deviates more, and we attributed this effect to the excursions when the environment is in the zero state. We also described more subtle non-linear effects, such as a phase in which the non-zero environment states that are below average dominate the mean, or a locally maximal deviation when a controller mitigates the effect of excursions. In the literature, the effect of modulated degradation was recently described in Keizer et al. (2019), Holehouse et al. (2020). Holehouse et al. investigated the effect of extrinsic noise in the reaction rates on an autoregulatory gene expression system. Focusing on the shape of the stationary distribution of the subsystem, they found that extrinsic noise in the degradation rate can help explore the second mode of the deterministically bistable system resulting in a bimodal distribution for a range of noise strengths. They also found noise induced bimodality. The effect of modulated degradation on the mean was explicitly studied by Keizer et al. for gene expression models with and without feedback using lognormally distributed slow extrinsic noise. The authors obtained that the mean increases by up to $10\%$ for a moderately increased coefficient of variation. In contrast, we studied the deviation for increased time scale separation between the environment and the embedded birth-death system and found that much larger increases are possible.

Since protein dilution rather than active degradation can be a primary environmental noise source, we compared our case study E1 with a model of dilution due to cell division Beentjes et al. (2020). In both models, the stationary mean increased compared to the respective reference model. Beentjes et al. consider this increase of the mean as small and irrelevant in their model, which is the case when *N* of the Erlang distribution is large. Instead, they bring to attention that the negative binomial stationary distribution of a birth-death model with geometrically distributed bursts persists under the influence of the environment, with a changed interpretation of the parameters. In contrast, with the environment speed as an additional degree of freedom we obtain large relative increases which we consider relevant. In addition, the comparison with a model that employed an Erlang distributed interjump time in the environment suggests to extend our approach from Markov to semi-Markov environments. Furthermore, this comparison showed that we can incorporate the instantaneous decays as long as the change of the mean can be expressed by a linear relation.

Models on continuous state spaces, e.g., Langevin equations, can often handle rates that become temporarily negative. However, in Holehouse et al. (2020), the approximating Langevin equations resulted in negative probabilities. Similarly, we found that our method can be sensitive to temporarily negative rates when summands in Eq. (13) get negative. This can prohibit rate linearization. When we compute ESME numerically, we need a truncation of the environment to a finite state space. For this, the finite state projection method (Bronstein and Koeppl 2018; Munsky and Khammash 2006) or other tools (Wolf et al. 2010) can be employed.

In the experimental context, we suggest that the effect of an environmental embedding can be distinguished from the Q.SS model with averaged environment via a characteristic fingerprint at the bulk level. Single stationary mean values are hardly informative to distinguish between both. However, functional dependencies over system parameters can look qualitatively different for a Q.SS model compared to an extension via an environmental embedding. In this work, we mostly investigated the time scale separation as a system parameter and saw a characteristic qualitative deviation. While the Q.SS model remained constant, the environment model generated a dependency with phases of distinct functional behaviour. It is hard to modify the time scale separation experimentally. However, other system parameters, such as volume, temperature, inducer concentration or time, can yield dependencies that achieve a phase change. In this way, the effect of the environment could be seen at the bulk level.

Reduction techniques for the stochastic simulation of chemical reaction networks can have difficulties in capturing the asymptotic mean correctly (Bronstein 2020, p.70). Our approach might assist in detecting the parameter regimes in which approximate simulations succeed. On the one hand, ESME can replace the Monte Carlo approximations. On the other hand, our quantification of environmental shares can permit insight into the failure mode of the approximate simulation or model reduction technique. The knowledge of the exact stationary mean can also tune approximation methods towards capturing the asymptotic mean correctly.

In future work, we seek to derive expressions for the stationary variance and generalize the expressions to a semi-Markov environment, analogously to Falin (2008), and to environments on continuous state space.

## Figures and Tables

**Fig. 7:**
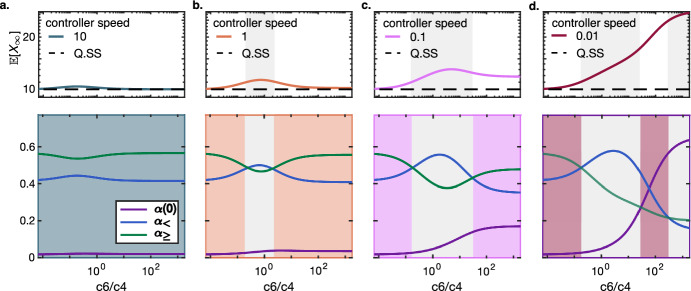
The controller $U$ becomes slower from **a**–**d**. Parameters were $c_5= 1, c_6= 1, c_1/c_2= 10, c_3/c_4= 4$. For small $c_6/c_4$ the stationary mean saturates to the Q.SS mean $c_1c_5/c_2c_6= 10$ independent. The saturation level for large $c_6/c_4$ depends on the speed $c_2$ of $U$. For fast enough $U$ a local maximum appears. For slower $U$ the saturation level increases and the contribution $\alpha (0)$ dominates more and more

**Fig. 8:**
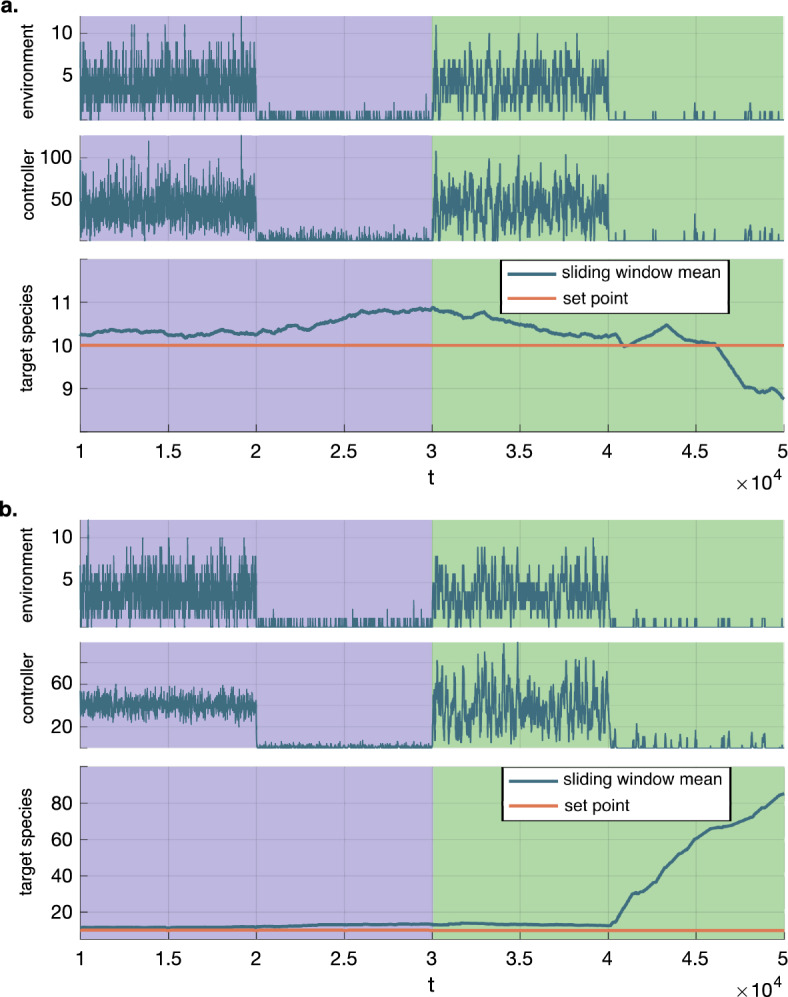
Trajectories for the environment E2 with controller. Environment $Z$ and controller $U$ are shown in plain form, while $X$ is depicted as a moving average with window length $t = 10,000$ to match the statement about the stationary mean. A burn-in time of $t = 10,000$ was used. The environment progresses through four stages of different mean and speed. **a** A fast controller ($c_2/c_6 = 10$) keeps the target species near the setpoint, on average, as the environment changes. According to the theory the deviation stays within $10\%$ of the setpoint. In the last phase a higher deviation indicates that stationarity is not reached within the window length. The drop below the setpoint is due to a low plateau value in the trajectory that would be counter-balanced by larger plateau values in the long run. **b** A slow controller ($c_2/c_6 = 0.1$) achieves low accuracy for the critical stage of low and slow environment (last phase). Parameter values were ${\mathbb {E}}[X_\infty ^{\mathrm {Q.SS}}] = {\mathbb {E}}[U] = 10, c_6= 1$ and in the four phases the four pairs $({\mathbb {E}}[Z], c_4) = (4, 3), (0.1, 3), (4, 0.03), (0.1, 0.03)$ were used

## Appendix A Proofs of Proposition 1 and the main result (ESME)

### Proof of Proposition 1

#### Proof

We verify the stationarity condition for the embedded chain.

$$\begin{aligned} \sum _{{z}\in {\mathcal {Z}}} K({z}', {z})\tilde{{\pi }}({z}) = \sum _{{z}\ne {z}'} \frac{{\Lambda }({z}', {z})}{{\Lambda }_0({z})} {\pi }({z}){\Lambda }({z}) = - {\Lambda }({z}', {z}') {\pi }({z}') = {\Lambda }_0({z}'){\pi }({z}') = \tilde{{\pi }}({z}'). \end{aligned}$$

$$\square $$

### Proofs of Proposition 2 and Theorem 3 (ESME)

In the proofs we make use of the following lemma.

#### Lemma 4

Let *T* be exponentially distributed with parameter $\mu > 0$ and suppose that all eigenvalues of ${\textbf{A}}$ have non-negative real part. Then $\mu {{\,\mathrm{{\textbf{I}}}\,}}+ {\textbf{A}}$ is invertible and

(i) $$\begin{aligned} {\mathbb {E}}[e^{-{\textbf{A}} T}] = \mu (\mu {{\,\mathrm{{\textbf{I}}}\,}}+ {\textbf{A}})^{-1} \end{aligned}$$
(ii) $$\begin{aligned} {\mathbb {E}}[\int _0^T e^{-{\textbf{A}} t}\,\mathrm dt] = (\mu {{\,\mathrm{{\textbf{I}}}\,}}+ {\textbf{A}})^{-1} \end{aligned}$$
(iii) $$\begin{aligned} {\mathbb {E}}[\int _0^T \int _0^t e^{-{\textbf{A}} s} \,\mathrm ds\,\mathrm dt] = \mu ^{-1}(\mu {{\,\mathrm{{\textbf{I}}}\,}}+ {\textbf{A}})^{-1} \end{aligned}$$

#### Proof of the lemma

$$\begin{aligned} {\mathbb {E}}[e^{-{\textbf{A}} T}]&= \int _0^\infty \mu e^{-\mu t} e^{-{\textbf{A}} t}\,\mathrm dt = \mu (\mu {{\,\mathrm{{\textbf{I}}}\,}}+ {\textbf{A}})^{-1}\\ {\mathbb {E}}[\int _0^T e^{-{\textbf{A}} t}\,\mathrm dt]&= \int _0^\infty \mu e^{-\mu \tau }\int _0^\tau e^{-{\textbf{A}} t}\,\mathrm dt\,\mathrm d\tau = \int _0^\infty e^{-{\textbf{A}} t} \int _t^\infty \mu e^{-\mu \tau }\,\mathrm d\tau \,\mathrm dt \\ {}&= \int _0^\infty e^{-{\textbf{A}} t} e^{-\mu t}\,\mathrm ds = (\mu {{\,\mathrm{{\textbf{I}}}\,}}+ {\textbf{A}})^{-1}\\ {\mathbb {E}}[\int _0^T \int _0^t e^{-{\textbf{A}} s} \,\mathrm ds\,\mathrm dt]&= \int _0^\infty \mu e^{-\mu \tau }\int _0^\tau \int _0^t e^{-{\textbf{A}} s}\,\mathrm ds \,\mathrm dt \,\mathrm d\tau \\ {}&= \int _0^\infty e^{-{\textbf{A}} s} \int _s^\infty \int _t^\infty \mu e^{-\mu \tau }\,\mathrm d\tau \,\mathrm dt \,\mathrm ds\\&= \int _0^\infty e^{-{\textbf{A}} s} \int _s^\infty e^{-\mu t}\,\mathrm dt \,\mathrm ds = \int _0^\infty e^{-{\textbf{A}} s} \mu ^{-1}e^{-\mu s}\,\mathrm ds \\&= \mu ^{-1}(\mu {{\,\mathrm{{\textbf{I}}}\,}}+ {\textbf{A}})^{-1} \end{aligned}$$

$$\square $$

#### Proof of proposition 2

$$\begin{aligned} {\textbf{x}}({z})&= {\mathbb {E}}[{{\textbf {Y}}}(\tau _{n+1})\vert {Z}(\tau _n) = {z}]\\&= {\mathbb {E}}[e^{-{{\textbf {A}}}({z})(\tau _{n+1}-\tau _n)}\vert {Z}(\tau _n) = {z}] {\mathbb {E}}[{{\textbf {Y}}}(\tau _n)\vert {Z}(\tau _n) = {z}] \\&\quad + {\mathbb {E}}[\int _{0}^{\tau _{n+1}-\tau _n} e^{-{{\textbf {A}}}({z})t}\,\mathrm dt \vert {Z}(\tau _n) = {z}] {{\textbf {b}}}({z})\\&={\Lambda }_0({z})({\Lambda }_0({z}){{\,\mathrm{{\textbf{I}}}\,}}+ {{\textbf {A}}}({z}))^{-1}\sum _{{z}' \ne {z}} {\mathbb {P}}[{W}_{n-1} = {z}'\vert {W}_{n} = {z}] {\textbf{x}}({z}') + ({\Lambda }_0({z}){{\,\mathrm{{\textbf{I}}}\,}}\\&\quad + {{\textbf {A}}}({z}))^{-1}{{\textbf {b}}}({z})\\&={\Lambda }_0({z})({\Lambda }_0({z}){{\,\mathrm{{\textbf{I}}}\,}}+ {{\textbf {A}}}({z}))^{-1}\sum _{{z}' \ne {z}} K({z}, {z}')\frac{\tilde{{\pi }}({z}')}{\tilde{{\pi }}({z})} {\textbf{x}}({z}') + ({\Lambda }_0({z}){{\,\mathrm{{\textbf{I}}}\,}}+ {{\textbf {A}}}({z}))^{-1}{{\textbf {b}}}({z})\\&={\Lambda }_0({z})({\Lambda }_0({z}){{\,\mathrm{{\textbf{I}}}\,}}+ {{\textbf {A}}}({z}))^{-1}\sum _{{z}' \ne {z}} {\Lambda }({z}, {z}')\frac{{\pi }({z}')}{{\pi }({z}){\Lambda }_0({z})} {\textbf{x}}({z}') + ({\Lambda }_0({z}){{\,\mathrm{{\textbf{I}}}\,}}+ {{\textbf {A}}}({z}))^{-1}{{\textbf {b}}}({z})\\&=({\Lambda }_0({z}){{\,\mathrm{{\textbf{I}}}\,}}+ {{\textbf {A}}}({z}))^{-1}\sum _{{z}' \ne {z}} {\Lambda }({z}, {z}')\frac{{\pi }({z}')}{{\pi }({z})} {\textbf{x}}({z}') + ({\Lambda }_0({z}){{\,\mathrm{{\textbf{I}}}\,}}+ {{\textbf {A}}}({z}))^{-1}{{\textbf {b}}}({z}) \end{aligned}$$

Hence,

$$\begin{aligned} ({\Lambda }_0({z}){{\,\mathrm{{\textbf{I}}}\,}}+ {{\textbf {A}}}({z})){\textbf{x}}({z}) = \sum _{{z}' \ne {z}} {\Lambda }({z}, {z}')\frac{{\pi }({z}')}{{\pi }({z})} {\textbf{x}}({z}') + {{\textbf {b}}}({z}), \end{aligned}$$

or upon adding of ${\Lambda }({z}, {z}) {\textbf{x}}({z})$:

$$\begin{aligned} {{\textbf {A}}}({z}){\textbf{x}}({z}) = \sum _{{z}' \in {\mathcal {Z}}} {\Lambda }({z}, {z}')\frac{{\pi }({z}')}{{\pi }({z})} {\textbf{x}}({z}') + {{\textbf {b}}}({z}). \end{aligned}$$

If detailed balance hold, we substitute

$$\begin{aligned} {\Lambda }({z}, {z}')\frac{{\pi }({z}')}{{\pi }({z})} ={\Lambda }({z}', {z}), \end{aligned}$$

yielding

$$\begin{aligned} {{\textbf {A}}}({z}){\textbf{x}}({z}) = \sum _{{z}' \in {\mathcal {Z}}} {\Lambda }^T({z}, {z}') {\textbf{x}}({z}') + {{\textbf {b}}}({z}). \end{aligned}$$

$$\square $$

#### Proof of Eq. (20)

The integrals evaluate to:

$$\begin{aligned}&{\mathbb {E}}[\int _{\tau _n}^{\tau _{n+1}} {{\textbf {Y}}}(t)\,\mathrm dt\vert {Z}(\tau _n) = {z}]\\&\quad = {\mathbb {E}}[\int _{0}^{\tau _{n+1}-\tau _n}e^{-{{\textbf {A}}}({z})(t)}\,\mathrm dt\vert {Z}(\tau _n) = {z}] {\mathbb {E}}[{{\textbf {Y}}}(\tau _n)\vert {Z}(\tau _n) = {z}] \\&\qquad + {\mathbb {E}}[\int _{0}^{\tau _{n+1}-\tau _n}\int _0^t e^{-{{\textbf {A}}}({z})s}\,\mathrm ds\,\mathrm dt \vert {Z}(\tau _n) = {z}] {{\textbf {b}}}({z})\\&\quad = ({\Lambda }_0({z}){{\,\mathrm{{\textbf{I}}}\,}}+ {{\textbf {A}}}({z}))^{-1} \sum _{{z}' \ne {z}} {\mathbb {P}}[{W}_{n-1} = {z}'\vert {W}_{n} = {z}] {\textbf{x}}({z}') \\&\qquad + {\Lambda }_0({z})^{-1}({\Lambda }_0({z}){{\,\mathrm{{\textbf{I}}}\,}}+ {{\textbf {A}}}({z}))^{-1}{{\textbf {b}}}({z})\\&\quad = \frac{{\textbf{x}}({z})}{{\Lambda }_0({z})} \end{aligned}$$

$$\square $$

#### Proof of theorem 3

$$\begin{aligned} {\mathbb {E}}[{{\textbf {X}}}_\infty ]&= \lim _{N \rightarrow \infty }\frac{\int _{0}^{\tau _N}{{\textbf {X}}}(t) \,\mathrm dt}{\tau _N} \\&=\frac{\lim _{N \rightarrow \infty }\frac{1}{N}\sum _{n= 0}^N \int _{\tau _n}^{\tau _{n+1}} {{\textbf {X}}}(t)\,\mathrm dt\sum _{{z}\in {\mathcal {Z}}} \mathbb {1}({Z}(\tau _n) = {z})}{\lim _{N \rightarrow \infty }\frac{1}{N}\sum _{n= 0}^N (\tau _{n+1}-\tau _n) \sum _{{z}\in {\mathcal {Z}}} \mathbb {1}({Z}(\tau _n) = {z})} \\&= \frac{\sum _{{z}\in {\mathcal {Z}}} \lim _{N \rightarrow \infty }\frac{1}{N}\sum _{n = 0}^N\mathbb {1}({Z}(\tau _n) = {z}) \int _{\tau _n}^{\tau _{n+1}} {{\textbf {X}}}(t)\,\mathrm dt}{\sum _{{z}\in {\mathcal {Z}}} \lim _{N \rightarrow \infty } \frac{1}{N}\sum _{n = 0}^N\mathbb {1}({Z}(\tau _n) = {z}) (\tau _{n+1}-\tau _n)} \\ {}&= \frac{\sum _{{z}\in {\mathcal {Z}}} {\mathbb {P}}[{Z}(\tau _n) = {z}] {\mathbb {E}}[\int _{\tau _n}^{\tau _{n+1}} {{\textbf {X}}}(t)\vert {Z}(\tau _n) = {z}]}{\sum _{{z}\in {\mathcal {Z}}} {\mathbb {P}}[{Z}(\tau _n) = {z}] {\mathbb {E}}[\tau _{n+1}-\tau _n \vert {Z}(\tau _n) = {z}]} \\&= \frac{\sum _{{z}\in {\mathcal {Z}}} \tilde{{\pi }}({z}) \frac{{\textbf{x}}({z})}{{\Lambda }_0({z})}}{\sum _{{z}\in {\mathcal {Z}}} \tilde{{\pi }}({z}) {\Lambda }_0({z})^{-1}}\\ {}&= \frac{\sum _{{z}\in {\mathcal {Z}}} {{\pi }}({z}) {{\textbf{x}}({z})}}{\sum _{{z}\in {\mathcal {Z}}} {{\pi }}({z})} \end{aligned}$$

$$\square $$

## Appendix B Details on case studies

### E1: Two-state death rate modulation

The birth rate is a birth-death process and the death rate is a two-state Markov process (random telegraph model), i.e., consider the CRN (see Fig. 2a)

B1

The stationary means of $Z_1$ and $Z_2$ are easily identified as

$$\begin{aligned} {\mathbb {E}}[Z_1] = \frac{c_1}{c_2}, \qquad {\mathbb {E}}[Z_2] = \frac{c_3}{c_3+c_4}. \end{aligned}$$

In this case, the dimension of the subsystem is $d= 1$. Then $A({z}_1, {z}_2) = c_6{z}_2$ and $b({z}_1, {z}_2) = c_5{z}_1$ are scalars. Since $Z_1$ only enters via the zero-order reactions, i.e., via *b*, we use the Q.SS assumption and set it to its mean. This reduces the environment to ${Z}= Z_2$ on the state space ${\mathcal {Z}}= \{0,1\}$. The generator of the environment is

$$\begin{aligned} {{\varvec{\Lambda }}}= \begin{bmatrix} -c_3&{} c_4\\ c_3&{} -c_4\end{bmatrix} \end{aligned}$$

with stationary distribution ${\pi } = {{\,\textrm{Bernoulli}\,}}(c_3/(c_3+ c_4))$. The expression of the stationary mean is then (Eq. (15))

B2 $$\begin{aligned} {\mathbb {E}}[X_\infty ] = \frac{c_1c_5(c_3+ c_4)}{c_2c_3c_6}\left( 1 + \frac{c_6c_4}{(c_3+ c_4)^2}\right) . \end{aligned}$$

We analyzed the behaviour of the stationary mean ${\mathbb {E}}[X_\infty ]$ as a function of the relative correlation time $c_6/(c_3+ c_4)$.

### E2: Birth-death modulator

In Eq. (B1), replace the two reactions ${{\,\textrm{R}\,}}_3, {{\,\textrm{R}\,}}_4$ by

B3 $$\begin{aligned} {{\,\textrm{R}\,}}_3, {{\,\textrm{R}\,}}_4: \qquad \emptyset \overset{c_3}{\longrightarrow } Z_2\overset{c_4}{\longrightarrow }\emptyset . \end{aligned}$$

The full CRN is visualized in Fig. 2b.

While the collection of rates ${{\textbf {A}}}, {{\textbf {b}}}$ of the $X$ dynamics remains the same, we make adjustments in the stationary mean state space, generator and stationary distribution of $Z_2$ accordingly:

$$\begin{aligned} {\mathbb {E}}[Z_1] = \frac{c_1}{c_2}, \qquad {\mathbb {E}}[Z_2] = \frac{c_3}{c_4}. \end{aligned}$$

The standard birth-death generator on state space ${\mathcal {Z}}= {\mathbb {N}}_0$ is given by

B4 $$\begin{aligned} {\Lambda }({z}, {z}') = {\left\{ \begin{array}{ll}c_3, &{} {z}= {z}' + 1\\ c_4{z}',&{} {z}= {z}' - 1\\ -(c_3+ c_4{z}'), &{} {z}= {z}' \end{array}\right. } \end{aligned}$$

with stationary distribution ${\pi } = {{\,\textrm{Poisson}\,}}(c_3/c_4)$. The expression of the stationary mean was already presented in Falin (2008) and O’Cinneide and Purdue (1986). Analogously to model E1, we analyzed the behaviour of the stationary mean ${\mathbb {E}}[X_\infty ]$ as a function of the relative correlation time $c_6/c_4$. For computational purposes, we truncate the state space ${\mathcal {Z}}$ to ${\mathcal {Z}}_N= \{0, 1, \dots , N\}$ with a large enough $N$. Here, $N= 99$ was used.

#### Leakage

Compared to model E2, we include a base degradation rate $\lambda _0$ for reaction ${{\,\textrm{R}\,}}_6$, i.e., the propensity of reaction ${{\,\textrm{R}\,}}_6$ is ${f}(x, z) = (c_6z + \lambda _0)x$. This altered ${{\textbf {A}}}$, i.e., ${{\textbf {A}}}({z}) = c_6z + \lambda _0$. The Fig. 5 indicates four regimes, in particular the three phases of the model E2 analysis (Fig. 2e) persist. The fourth phase with largest $\tau _Z/\tau _X$ reaches saturation.

### E3: Mutable synthesis of the modulator

We modified reaction ${{\,\textrm{R}\,}}_3$ of model E2, see Eqs. (B1) and (B3), to

B5

Then the two-dimensional environment ${Z}= (Z_2,Z_3) \in {\mathbb {N}}\times \{0,1\}$ has a stationary distribution that is expressed via the confluent hypergeometric function. Expressions for ${\mathbb {P}}[Z_2= m, Z_3= 0]$ and ${\mathbb {P}}[Z_2= m, Z_3= 1]$ were derived analogously to Peccoud and Ycart (1995) by expanding the generating function given therein. Define $a = \frac{c_7}{c_4}, b = \frac{c_7+ c_8}{c_4}, \mu = \frac{c_3}{c_4}$. Then it holds

$$\begin{aligned} {\mathbb {P}}[Z_2= m, Z_3= 0]&=\frac{\Gamma (b) \Gamma (m+a) (b-a)}{\Gamma (a)\Gamma (m+b+1) } {}_1F_1(m + a,m + b + 1,-\mu ) \frac{\mu ^m}{m!}\\ {\mathbb {P}}[Z_2= m, Z_3= 1]&= \frac{\Gamma (b) \Gamma (m+a + 1)}{\Gamma (a)\Gamma (m+b+1) } {}_1F_1(m + a + 1,m + b + 1,-\mu ) \frac{\mu ^m}{m!}. \end{aligned}$$

ESME was calculated numerically using Eq. (14) with truncation $N= 100$. Figure 6a shows the entry of the stationary mean into the degenerate regime as a function of the relative speed of the modulator $Z_3\leftrightarrow Z_3^*$. Figure 6b depicts the environmental shares for the slow and the fast modulator.

### Asymptotic behavior in a slow environment

Consider any of the models E1, E2, E3. Under the fixation of ${\mathbb {E}}[X_\infty ^\text {Q.SS}], \frac{c_5}{c_6},{\mathbb {E}}[Z_2], {\mathbb {E}}[Z_3]$ and $\tau _Z/\tau _S$ the stationary mean only depends on the relative time scale $\tau _Z/\tau _X$. Hence in the following we fix $\tau _X = c_6^{-1} = 1$. Then, $\tau _Z/\tau _X \rightarrow \infty$ is equivalent to $c_4\rightarrow 0$. By our main theorem (3), we obtain ${\mathbb {E}}[X_\infty ] = {\mathcal {O}}({\pi }(0){\textbf{x}}(0))$ for $c_4\rightarrow 0$ in the models 1a, 1b and ${\mathbb {E}}[X_\infty ] = {\mathcal {O}}({\pi }(0,0){\textbf{x}}(0,0) + {\pi }(0,1){\textbf{x}}(0,1))$ for model E3. By definition ${\textbf{x}}(0) = {\mathbb {E}}[X(\tau _{n+1})\vert Z_2(\tau _n) = 0]$. The function $t \mapsto {\mathbb {E}}[X(t)\vert Z_2(\tau _n) = 0]$ progresses affine-linearly in time. For very slow $Z_2$ the excursions are long and the base value at $\tau _n$, from which they depart, is small compared to the value they reach at $\tau _{n+1}$. We thus neglect the base value to obtain a linear progression with constant slope $c_5{\mathbb {E}}[Z_1]$, yielding

$$\begin{aligned} x(0) = {\mathcal {O}}(c_5{\mathbb {E}}[Z_1]{\mathbb {E}}[\tau _{n+1} - \tau _n\vert Z_2(\tau _n) = 0]) = {\mathcal {O}}\left( \frac{c_1c_5}{c_2c_3}\right) . \end{aligned}$$

In total we obtain for the stationary mean of models E1, E2

$$\begin{aligned} {\mathbb {E}}[X_\infty ] = {\mathcal {O}}\left( \frac{c_1c_5}{c_2c_3}{\mathbb {P}}[Z_2= 0]\right) . \end{aligned}$$

Since ${\mathbb {P}}[Z_2= 0]$ only depends on the mean ${\mathbb {E}}[Z_2]$ which we keep fixed, the parameter $c_3$ which scaled linearly with $c_4$ dominates the asymptotic behaviour $c_4\rightarrow 0$. More precisely, we obtain

$$\begin{aligned} \lim _{c_4\rightarrow 0}\frac{{\mathbb {E}}[X_\infty ]}{c_4^{-1}} = {\left\{ \begin{array}{ll} c_5{\mathbb {E}}[Z_1]\frac{(1-{\mathbb {E}}[Z_2])^2}{{\mathbb {E}}[Z_2]}, &{}Z_2\text { random telegraph}\\ c_5{\mathbb {E}}[Z_1]\frac{\exp (-{\mathbb {E}}[Z_2])}{{\mathbb {E}}[Z_2]}, &{}Z_2\text { birth-death} \end{array}\right. }. \end{aligned}$$

For model E3 the two states $(Z_2, Z_3) = (0,0)$ and (0, 1) contribute for $c_4\rightarrow 0$. After setting up the recursion that couples *x*(0, 0) and *x*(0, 1), see next paragraph, we obtain

$$\begin{aligned} \lim _{c_4\rightarrow 0}\frac{{\mathbb {E}}[X_\infty ]}{c_4^{-1}} = c_5{\mathbb {E}}[Z_1]\left( \frac{{\mathbb {P}}[Z_2= 0]}{{\mathbb {E}}[Z_2]} + \frac{c_4}{c_8}\frac{{\mathbb {P}}[Z_2= 0, Z_3= 0]}{1-{\mathbb {E}}[Z_3]}\right) \end{aligned}$$

Note that ${\mathbb {P}}[Z_2= 0]$ and ${\mathbb {P}}[Z_2= 0, Z_3= 0]$ only depend on the relative rates $\frac{c_7}{c_4}, \frac{c_8}{c_4}, \frac{c_3}{c_4}$, which were fixed, i.e., the time scale of the joint environment $(Z_2, Z_3)$ was varied. The inverse proportional dependence on $c_4$ for models E1–E3 is visualized in Fig. 2c, f, i.

For $c_4\rightarrow 0$ we compute the stationary mean under assumption that all terms in Eq. (13) vanish except for ${z}= (0,0), (0,1)$. The recursion Eq. (10) for the two states (0, 0) and (0, 1) read

$$\begin{aligned} 0&= -c_7{\pi }(0,0){\textbf{x}}(0,0) + c_8{\pi }(0,1) {\textbf{x}}(0,1) + c_4{\pi }(1,0) {\textbf{x}}(1,0)+ \frac{c_5c_1}{c_2} {\pi }(0,0)\\ 0&= -(c_8+ c_3) {\pi }(0,1){\textbf{x}}(0,1) + c_7{\pi }(0,0) {\textbf{x}}(0,0) + c_4{\pi }(1,1){\textbf{x}}(1,1)+\frac{c_5c_1}{c_2}{\pi }(0,1) \end{aligned}$$

By assumption,

$$\begin{aligned} \max \{{\pi }(1,1){\textbf{x}}(1,1), {\pi }(1,0){\textbf{x}}(1,0)\} \ll \min \{{\pi }(0,1){\textbf{x}}(0,1), {\pi }(0,0){\textbf{x}}(0,0)\} \end{aligned}$$

so we can set ${\pi }(1,1){\textbf{x}}(1,1) = {\pi }(1,0){\textbf{x}}(1,0) = 0$. Then the 2 dimensional linear system has the solution

$$\begin{aligned} {\pi }(0,0){\textbf{x}}(0,0)&= \frac{c_5c_1}{c_2}\cdot \left( \frac{c_8+ c_3}{c_3c_7}{\pi }(0,0) + \frac{c_8}{c_3c_7}{\pi }(0,1)\right) \\ {\pi }(0,1){\textbf{x}}(0,1)&=\frac{c_5c_1}{c_2}\cdot \frac{{\pi }(0,0) + {\pi }(0,1)}{c_3} \end{aligned}$$

which by ${\mathbb {E}}[X_\infty ] = {\pi }(0,0){\textbf{x}}(0,0) + {\pi }(0,1){\textbf{x}}(0,1)$ yields the result for $\lim _{c_4\rightarrow 0}{\mathbb {E}}[X_\infty ]c_4$.

### E2 + controller: a correlated environment

Compared to environment E2, we replaced the reaction ${{\,\textrm{R}\,}}_1$ in Eqs. (B1), (B3) by

$$\begin{aligned} {{\,\textrm{R}\,}}_1: \qquad Z_2\overset{c_1}{\longrightarrow }&Z_2+ Z_1\end{aligned}$$

In order to apply our general framework we regard $Z_2$ as the one-dimensional environment and $(Z_1, X)$ as the modulated linear CRN. Then ${\mathcal {Z}}= {\mathbb {N}}_0$ and ${\Lambda }$ is the generator of a birth-death process with birth rate $c_3$ and death rate $c_4$. Detailed balance is satisfied by Poisson-distributed ${\pi }$ with parameter $c_3/c_4$. For the dynamics of ${{\textbf {Y}}}$ we obtain

$$\begin{aligned} {{\textbf {A}}}({z})&= \begin{bmatrix} c_2&{} 0\\ -c_5&{} c_6{z}\end{bmatrix}\\ {{\textbf {b}}}({z})&=\begin{bmatrix} c_1{z}\\ 0 \end{bmatrix}. \end{aligned}$$

We fix $c_6, c_5$ and the means $\frac{c_1}{c_2}, \frac{c_3}{c_4}$. For different speeds $c_2$ of $Z_1$ we let the speed $c_4$ of $Z_2$ vary, see Fig. 2k. For large $c_4$ the stationary mean saturates to the Q.SS mean $c_1c_5/c_2c_6= 10$ independent of $c_2$. The saturation level for small $c_4$ depends on the speed $c_2$ of $Z_1$. For fast enough $Z_1$ a local maximum appears. For slower $Z_1$ the saturation level increases. The stationary mean curve for small $c_2$ resembles the leakage case displaying the same four phases.

The birth-death process $X$ in a correlated environment showed a local maximum in the phase analysis. Here we provide details on the shares $\alpha (0), \alpha _{<{\bar{{z}}}}, \alpha _{\ge {\bar{{z}}}}$, see Fig. 7. Furthermore, Fig. 8 provides example trajectories for a fast and a slow controller.

### Stochastic toggle switch in a random environment

Given the model reactions, the dynamics of ${\mathbb {E}}[\textbf{X}_{\infty }]$ can be described with the following reaction matrices:

$$\begin{aligned} {{\textbf {A}}}({{\textbf {z}}})&= \begin{bmatrix} c_6 z_2 &{} b_1 z_1\\ b_2 z_1 &{} c_6 z_2 \end{bmatrix},\\ {{\textbf {b}}}({{\textbf {z}}})&=\begin{bmatrix} a_1 z_1 \\ a_2 z_1 \end{bmatrix}. \end{aligned}$$

The generator used here is the one corresponding to two jointly independent birth-death processes, and it is of the form

$$\begin{aligned} {{\varvec{\Lambda }}}= {{\,\mathrm{{\textbf{I}}}\,}}_1 \otimes {{\varvec{\Lambda }}}_2 + {{\varvec{\Lambda }}}_1 \otimes {{\,\mathrm{{\textbf{I}}}\,}}_2 \end{aligned}$$

with ${{\,\mathrm{{\textbf{I}}}\,}}_i \in {\mathbb {R}}^{N_i \times N_i}$ the identity matrices for state space truncation $Z_i \in \{0,1, \dots , N_i - 1\}$ and ${{\varvec{\Lambda }}}_i$ analogous to Eq. (B4). Here, $N_1 = N_2 = 50$ was used.

To pick the model parameters for a given $r_0$ ratio, we fixed all parameters besides the rates $a_1$ and $a_2$ and computed those rates from the equation ${{\textbf {b}}}({z}) = {{\textbf {A}}}({z}){\mathbb {E}}[\textbf{X}^\mathrm {Q.SS}_{\infty }]$.

### Stochastic oscillator in a random environment

Given the model reactions, the dynamics of ${\mathbb {E}}[\textbf{X}_{\infty }]$ can be described with the following reaction matrices:

$$\begin{aligned} {{\textbf {A}}}({{\textbf {z}}})&= \begin{bmatrix} c_6 z_2 &{} b_1 z_1\\ -b_2 z_1 &{} c_6 z_2 \end{bmatrix}\\ {{\textbf {b}}}({{\textbf {z}}})&=\begin{bmatrix} a_1 z_1 \\ 0 \end{bmatrix}. \end{aligned}$$

The generator used here is the one corresponding to two jointly independent birth-death processes, and it is of the form

$$\begin{aligned} {{\varvec{\Lambda }}}= {{\,\mathrm{{\textbf{I}}}\,}}_1 \otimes {{\varvec{\Lambda }}}_2 + {{\varvec{\Lambda }}}_1 \otimes {{\,\mathrm{{\textbf{I}}}\,}}_2 \end{aligned}$$

with ${{\,\mathrm{{\textbf{I}}}\,}}_i \in {\mathbb {R}}^{N_i \times N_i}$ the identity matrices for state space truncation $Z_i \in \{0,1, \dots , N_i - 1\}$ and ${{\varvec{\Lambda }}}_i$ analogous to Eq. (B4). Here, $N_1 = N_2 = 50$ was used.

## Appendix C Details on linearization

We matched the linearized and the Hill function models of repression as follows. Namely, first in $g_1$, we obtained

$$\begin{aligned} a_1= & {} {\tilde{g}}_1({\mathbb {E}}[X^\mathrm {Q.SS}_{2,\infty }]) - {\tilde{g}}_1'({\mathbb {E}}[X^\mathrm {Q.SS}_{2,\infty }]){\mathbb {E}}[X^\mathrm {Q.SS}_{2,\infty }] \\ b_1= & {} - {\tilde{g}}_1'({\mathbb {E}}[X^\mathrm {Q.SS}_{2,\infty }]) \end{aligned}$$

and then in ${\tilde{g}}_1$ we expressed $k_1$ and $K_{A_1}$ in terms of $a_1$ and $b_1$ (analogously, the same is done for $k_2$ and $K_{A_2}$):

$$\begin{aligned} {\left\{ \begin{array}{ll} k_1 = \frac{b_1 (\frac{a_1}{b_1} - {{\bar{x}}}_2)^2}{\frac{a_1}{b_1} - 2{{\bar{x}}}_2}\\ K_A = \frac{a_1}{b_1} - 2{{\bar{x}}}_2\\ \end{array}\right. }. \end{aligned}$$

The expansion point was chosen from the linearized model, in which it was the unique stable fixed point. To match this situation with the Hill function model, we restricted our study to the regime, where the corresponding deterministic model is monostable and the stochastic model is in the unimodal regime.

### Limitiations

Here, we elaborate on our understanding of the limitations of the linearization in the switch and oscillator case studies.

- First Limitation: As soon as $X_1$ or $X_2$ get too large, the propensities of the reactions ${{\,\textrm{R}\,}}_5, {{\,\textrm{R}\,}}_6$ become negative. This can happen for any choice of non-negative rate constants. Consequently, the linear dynamics Eq. (7) conditioned on ${Z}= {z}$ can saturate at a state with a negative value for a subsystem component. This can, for large enough waiting time, cause the integral in the share Eq. (21) to become negative, and can be detected by checking for negative shares. Eventually, this can result in a negative stationary mean. In the toggle switch case study we checked that in the portrayed parameter range (i) no share is negative and that (ii) the total share of those environmental states for which the saturation point has a negative component, is sufficiently small.
- Second Limitation: Negative eigenvalues of the matrix ${{\textbf {A}}}({z})$ can arise for some values of ${z}$. This means that the linear dynamics Eq. (7) conditioned on ${Z}= {z}$ gets unstable. If the waiting time in the state is short enough or, equivalently, ${\Lambda }_0({z})$ is large enough, then the unstable dynamics is mitigated and the formulas in Lemma 4 remain valid. This was not a limitation in the toggle switch case study due to a large enough $c_1$.
- Third Limitation: The matrix ${{\textbf {A}}}- {\Lambda }\otimes {{\,\mathrm{{\textbf{I}}}\,}}$ in Eq. (14) can get singular. We observed this in the toggle switch example for the choice $c_4= 0.015$ and other parameters that determine ${{\textbf {A}}}- {\Lambda }\otimes {{\,\mathrm{{\textbf{I}}}\,}}$ as in Fig. 4, causing an asymptote in ${\mathbb {E}}[X_{1,\infty }]$ and ${\mathbb {E}}[X_{2,\infty }]$. This parameter choice was, however, already excluded by the first limitation.
